# Manifestations of Alzheimer’s disease genetic risk in the blood are evident in a multiomic analysis in healthy adults aged 18 to 90

**DOI:** 10.1038/s41598-022-09825-2

**Published:** 2022-04-12

**Authors:** Laura Heath, John C. Earls, Andrew T. Magis, Sergey A. Kornilov, Jennifer C. Lovejoy, Cory C. Funk, Noa Rappaport, Benjamin A. Logsdon, Lara M. Mangravite, Brian W. Kunkle, Eden R. Martin, Adam C. Naj, Nilüfer Ertekin-Taner, Todd E. Golde, Leroy Hood, Nathan D. Price, Alzheimer’s Disease Genetics Consortium, Erin Abner, Perrie M. Adams, Marilyn S. Albert, Roger L. Albin, Mariet Allen, Alexandre Amlie-Wolf, Liana G. Apostolova, Steven E. Arnold, Sanjay Asthana, Craig S. Atwood, Clinton T. Baldwin, Robert C. Barber, Lisa L. Barnes, Sandra Barral, Thomas G. Beach, James T. Becker, Gary W. Beecham, Duane Beekly, David Bennett, Eileen H. Bigio, Thomas D. Bird, Deborah Blacker, Bradley F. Boeve, James D. Bowen, Adam Boxer, James R. Burke, Jeffrey M. Burns, Will Bush, Mariusz Butkiewicz, Joseph D. Buxbaum, Nigel J. Cairns, Laura B. Cantwell, Chuanhai Cao, Chris S. Carlson, Cynthia M. Carlsson, Regina M. Carney, Helena C. Chui, Paul K. Crane, David H. Cribbs, Elizabeth A. Crocco, Michael L. Cuccaro, Philip L. De Jager, Charles DeCarli, Malcolm Dick, Dennis W. Dickson, Beth A. Dombroski, Rachelle S. Doody, Ranjan Duara, Nilufer Ertekin-Taner, Denis A. Evans, Kelley M. Faber, Thomas J. Fairchild, Kenneth B. Fallon, David W. Fardo, Martin R. Farlow, Lindsay A. Farrer, Steven Ferris, Tatiana M. Foroud, Matthew P. Frosch, Douglas R. Galasko, Marla Gearing, Daniel H. Geschwind, Bernardino Ghetti, John R. Gilbert, Alison M. Goate, Robert C. Green, John H. Growdon, Jonathan Haines, Hakon Hakonarson, Ronald L. Hamilton, Kara L. Hamilton-Nelson, Lindy E. Harrell, Lawrence S. Honig, Ryan M. Huebinger, Matthew J. Huentelman, Christine M. Hulette, Bradley T. Hyman, Gail P. Jarvik, Lee-Way Jin, Gyungah R. Jun, M. Ilyas Kamboh, Anna Karydas, Mindy J. Katz, Jeffrey A. Kaye, C. Dirk Keene, Ronald Kim, Neil W. Kowall, Joel H. Kramer, Walter A. Kukull, Brian W. Kunkle, Amanda B. Kuzma, Frank M. LaFerla, James J. Lah, Eric B. Larson, James B. Leverenz, Allan I. Levey, Andrew P. Lieberman, Richard B. Lipton, Kathryn L. Lunetta, Constantine G. Lyketsos, John Malamon, Daniel C. Marson, Eden R. Martin, Frank Martiniuk, Deborah C. Mash, Eliezer Masliah, Richard Mayeux, Wayne C. McCormick, Susan M. McCurry, Andrew N. McDavid, Ann C. McKee, Marsel Mesulam, Bruce L. Miller, Carol A. Miller, Joshua W. Miller, Thomas J. Montine, John C. Morris, Shubhabrata Mukherjee, Amanda J. Myers, Adam C. Naj, Sid O’Bryant, John M. Olichney, Joseph E. Parisi, Henry L. Paulson, Margaret A. Pericak-Vance, William R. Perry, Elaine Peskind, Ronald C. Petersen, Aimee Pierce, Wayne W. Poon, Huntington Potter, Liming Qu, Joseph F. Quinn, Ashok Raj, Murray Raskind, Eric M. Reiman, Barry Reisberg, Joan S. Reisch, Christiane Reitz, John M. Ringman, Erik D. Roberson, Ekaterina Rogaeva, Howard J. Rosen, Roger N. Rosenberg, Donald R. Royall, Mark A. Sager, Mary Sano, Andrew J. Saykin, Gerard D. Schellenberg, Julie A. Schneider, Lon S. Schneider, William W. Seeley, Susan Slifer, Amanda G. Smith, Yeunjoo Song, Joshua A. Sonnen, Salvatore Spina, Peter St George-Hyslop, Robert A. Stern, Russell H. Swerdlow, Mitchell Tang, Rudolph E. Tanzi, John Q. Trojanowski, Juan C. Troncoso, Debby W. Tsuang, Otto Valladares, Vivianna M. Van Deerlin, Linda J. Van Eldik, Jeffery Vance, Badri N. Vardarajan, Harry V. Vinters, Jean Paul Vonsattel, Li-San Wang, Sandra Weintraub, Kathleen A. Welsh-Bohmer, Patrice Whitehead, Kirk C. Wilhelmsen, Jennifer Williamson, Thomas S. Wingo, Randall L. Woltjer, Clinton B. Wright, Chuang-Kuo Wu, Steven G. Younkin, Chang-En Yu, Lei Yu, Yi Zhao

**Affiliations:** 1 Institute for Systems Biology, Seattle, WA USA; 2 Sage Bionetworks, Seattle, WA USA; 3 Thorne HealthTech, New York, NY USA; 4 John P. Hussman Institute for Human Genomics, University of Miami Miller School of Medicine, Miami, FL USA; 5 Dr. John T. Macdonald Foundation Department of Human Genetics, University of Miami Miller School of Medicine, Miami, FL USA; 6 Department of Biostatistics, Epidemiology and Informatics, University of Pennsylvania Perelman School of Medicine, Philadelphia, PA USA; 7 Department of Pathology and Laboratory Medicine, University of Pennsylvania Perelman School of Medicine, Philadelphia, PA USA; 8 Department of Neurology, Mayo Clinic, Jacksonville, FL USA; 9 Department of Neuroscience, Mayo Clinic, Jacksonville, FL USA; 10 Department of Neuroscience, College of Medicine, McKnight Brain Institute, Center for Translational Research in Neurodegenerative Disease University of Florida, Gainesville, FL USA; 11 Providence St. Joseph Health, Renton, WA USA; 12 Department of Epidemiology, College of Public Health, Sanders-Brown Center on Aging, University of Kentucky, Lexington, KY USA; 13 Department of Psychiatry, University of Texas Southwestern Medical Center, Dallas, TX USA; 14 Department of Neurology, Johns Hopkins University, Baltimore, MD USA; 15 Department of Neurology, University of Michigan, Ann Arbor, MI USA; 16 Geriatric Research, Education and Clinical Center (GRECC), VA Ann Arbor Healthcare System (VAAAHS), Ann Arbor, MI USA; 17 Michigan Alzheimer Disease Center, Ann Arbor, MI USA; 18 Department of Pathology and Laboratory Medicine, Penn Neurodegeneration Genomics Center, University of Pennsylvania Perelman School of Medicine, Philadelphia, PA USA; 19 Department of Neurology, Radiology, Medical and Molecular Genetics, and Indiana Alzheimer’s Disease Center, Indiana University School of Medicine, Indianapolis, IN USA; 20 Department of Psychiatry, University of Pennsylvania Perelman School of Medicine, Philadelphia, PA USA; 21 Geriatric Research, Education and Clinical Center (GRECC), University of Wisconsin, Madison, WI USA; 22 Department of Medicine, University of Wisconsin, Madison, WI USA; 23 Wisconsin Alzheimer’s Disease Research Center, Madison, WI USA; 24 Department of Medicine (Genetics Program), Boston University, Boston, MA USA; 25 Department of Pharmacology and Neuroscience, University of North Texas Health Science Center, Fort Worth, TX USA; 26 Department of Neurological Sciences, Rush University Medical Center, Chicago, IL USA; 27 Department of Behavioral Sciences, Rush University Medical Center, Chicago, IL USA; 28 Rush Alzheimer’s Disease Center, Rush University Medical Center, Chicago, IL USA; 29 Taub Institute on Alzheimer’s Disease and the Aging Brain, Columbia University, New York, NY USA; 30 Gertrude H. Sergievsky Center, Columbia University, New York, NY USA; 31 Department of Neurology, Columbia University, New York, NY USA; 32 Civin Laboratory for Neuropathology, Banner Sun Health Research Institute, Phoenix, AZ USA; 33 Departments of Psychiatry, Neurology, and Psychology, University of Pittsburgh School of Medicine, Pittsburgh, PA USA; 34 National Alzheimer’s Coordinating Center, University of Washington, Seattle, WA USA; 35 Department of Pathology, Northwestern University Feinberg School of Medicine, Chicago, IL USA; 36 Cognitive Neurology and Alzheimer’s Disease Center, Northwestern University Feinberg School of Medicine, Chicago, IL USA; 37 VA Puget Sound Health Care System/GRECC, Seattle, WA USA; 38 Department of Neurology, University of Washington, Seattle, WA USA; 39 Department of Epidemiology, Harvard School of Public Health, Boston, MA USA; 40 Department of Psychiatry, Massachusetts General Hospital/Harvard Medical School, Boston, MA USA; 41 Department of Neurology, Mayo Clinic, Rochester, MN USA; 42 Swedish Medical Center, Seattle, WA USA; 43 Department of Neurology, University of California San Francisco, San Francisco, CA USA; 44 Department of Medicine, Duke University, Durham, NC USA; 45 University of Kansas Alzheimer’s Disease Center, University of Kansas Medical Center, Kansas City, KS USA; 46 Institute for Computational Biology, Department of Population & Quantitative Health Sciences, Case Western Reserve University, Cleveland, OH USA; 47 Department of Genetics and Genomic Sciences, Mount Sinai School of Medicine, New York, NY USA; 48 Department of Neuroscience, Mount Sinai School of Medicine, New York, NY USA; 49 Department of Psychiatry, Mount Sinai School of Medicine, New York, NY USA; 50 Department of Pathology and Immunology, Washington University, St. Louis, MO USA; 51 USF Health Byrd Alzheimer’s Institute, University of South Florida, Tampa, FL USA; 52 Fred Hutchinson Cancer Research Center, Seattle, WA USA; 53 Mental Health & Behavioral Science Service, Bruce W. Carter VA Medical Center, Miami, FL USA; 54 Department of Neurology, University of Southern California, Los Angeles, CA USA; 55 Department of Medicine, University of Washington, Seattle, WA USA; 56 Department of Neurology, University of California Irvine, Irvine, CA USA; 57 Department of Psychiatry and Behavioral Sciences, Miller School of Medicine, University of Miami, Miami, FL USA; 58 Department of Neurology, Center for Translational and Computational Neuroimmunology, Columbia University Medical Center, New York, NY USA; 59 Department of Neurology, University of California Davis, Sacramento, CA USA; 60 Institute for Memory Impairments and Neurological Disorders, University of California Irvine, Irvine, CA USA; 61 Baylor College of Medicine, Alzheimer’s Disease and Memory Disorders Center, Houston, TX USA; 62 Wien Center for Alzheimer’s Disease and Memory Disorders, Mount Sinai Medical Center, Miami Beach, FL USA; 63 Department of Internal Medicine, Rush Institute for Healthy Aging, Rush University Medical Center, Chicago, IL USA; 64 Department of Medical and Molecular Genetics, Indiana University, Indianapolis, IN USA; 65 Office of Strategy and Measurement, University of North Texas Health Science Center, Fort Worth, TX USA; 66 Department of Pathology, University of Alabama at Birmingham, Birmingham, AL USA; 67 Department of Biostatistics, Sanders-Brown Center on Aging, University of Kentucky, Lexington, KY USA; 68 Department of Neurology, Indiana University, Indianapolis, IN USA; 69 Department of Medicine (Biomedical Genetics), Boston University, Boston, MA USA; 70 Department of Epidemiology, BU School of Public Health, Boston, MA USA; 71 Department of Pathology, Boston University School of Medicine, Boston University, Boston, MA USA; 72 Department of Ophthalmology, Boston University School of Medicine, Boston University, Boston, MA USA; 73 Department of Psychiatry, New York University, New York, NY USA; 74 C.S. Kubik Laboratory for Neuropathology, Massachusetts General Hospital, Charlestown, MA USA; 75 Department of Neurosciences, University of California San Diego, La Jolla, CA USA; 76 Department of Pathology and Laboratory Medicine, Emory University, Atlanta, GA USA; 77 Emory Alzheimer’s Disease Center, Emory University, Atlanta, GA USA; 78 Neurogenetics Program, University of California Los Angeles, Los Angeles, CA USA; 79 Department of Pathology and Laboratory Medicine, Indiana University, Indianapolis, IN USA; 80 Department of Genetics and Genomic Sciences, Icahn School of Medicine at Mount Sinai, New York, NY USA; 81 Department of Neuroscience, Icahn School of Medicine at Mount Sinai, Ronald M. Loeb Center for Alzheimer’s Disease, New York, NY USA; 82 Division of Genetics, Department of Medicine and Partners Center for Personalized Genetic Medicine, Brigham and Women’s Hospital and Harvard Medical School, Boston, MA USA; 83 Department of Neurology, Massachusetts General Hospital/Harvard Medical School, Boston, MA USA; 84 Children’s Hospital of Philadelphia and Division of Human Genetics, Department of Pediatrics, The Perelman School of Medicine, Center for Applied Genomics, University of Pennsylvania, Philadelphia, PA USA; 85 Department of Pathology (Neuropathology), University of Pittsburgh, Pittsburgh, PA USA; 86 Department of Neurology, University of Alabama at Birmingham, Birmingham, AL USA; 87 Department of Surgery, University of Texas Southwestern Medical Center, Dallas, TX USA; 88 Neurogenomics Division, Translational Genomics Research Institute, Phoenix, AZ USA; 89 Department of Pathology, Duke University, Durham, NC USA; 90 Department of Genome Sciences, University of Washington, Seattle, WA USA; 91 Department of Medicine (Medical Genetics), University of Washington, Seattle, WA USA; 92 Department of Pathology and Laboratory Medicine, University of California Davis, Sacramento, CA USA; 93 Department of Psychiatry, University of Pittsburgh, Pittsburgh, PA USA; 94 Department of Human Genetics, University of Pittsburgh, Pittsburgh, PA USA; 95 Alzheimer’s Disease Research Center, University of Pittsburgh, Pittsburgh, PA USA; 96 Department of Neurology, Albert Einstein College of Medicine, New York, NY USA; 97 Department of Neurology, Oregon Health & Science University, Portland, OR USA; 98 Department of Neurology, Portland Veterans Affairs Medical Center, Portland, OR USA; 99 Department of Pathology, University of Washington, Seattle, WA USA; 100 Department of Pathology and Laboratory Medicine, University of California Irvine, Irvine, CA USA; 101 Department of Neurology, Boston University, Boston, MA USA; 102 Department of Pathology, Boston University, Boston, MA USA; 103 Department of Neuropsychology, University of California San Francisco, San Francisco, CA USA; 104 Department of Epidemiology, University of Washington, Seattle, WA USA; 105 Department of Neurobiology and Behavior, University of California Irvine, Irvine, CA USA; 106 Department of Neurology, Emory University, Atlanta, GA USA; 107 Kaiser Permanente Washington Health Research Institute, Seattle, WA USA; 108 Cleveland Clinic Lou Ruvo Center for Brain Health, Cleveland Clinic, Cleveland, OH USA; 109 Department of Pathology, University of Michigan, Ann Arbor, MI USA; 110 Department of Biostatistics, Boston University, Boston, MA USA; 111 Department of Psychiatry, Johns Hopkins University, Baltimore, MD USA; 112 Department of Medicine - Pulmonary, New York University, New York, NY USA; 113 Department of Neurology, University of Miami, Miami, FL USA; 114 Department of Pathology, University of California San Diego, La Jolla, CA USA; 115 School of Nursing Northwest Research Group on Aging, University of Washington, Seattle, WA USA; 116 Department of Neurology, Northwestern University Feinberg School of Medicine, Chicago, IL USA; 117 Weill Institute for Neurosciences, Memory and Aging Center, University of California, San Francisco, San Francisco, CA USA; 118 Department of Pathology, University of Southern California, Los Angeles, CA USA; 119 Department of Neurology, Washington University, St. Louis, MO USA; 120 Department of Laboratory Medicine and Pathology, Mayo Clinic, Rochester, MN USA; 121 Michigan Alzheimer’s Disease Center, University of Michigan, Ann Arbor, MI USA; 122 Department of Psychiatry and Behavioral Sciences, University of Washington School of Medicine, Seattle, WA USA; 123 Department of Neurology, University of Colorado School of Medicine, Aurora, CO USA; 124 Arizona Alzheimer’s Consortium, Phoenix, AZ USA; 125 Banner Alzheimer’s Institute, Phoenix, AZ USA; 126 Alzheimer’s Disease Center, New York University, New York, NY USA; 127 Department of Clinical Sciences, University of Texas Southwestern Medical Center, Dallas, TX USA; 128 Department of Epidemiology, Columbia University, New York, NY USA; 129 Department of Neurology, University of California Los Angeles, Los Angeles, CA USA; 130 Tanz Centre for Research in Neurodegenerative Disease, University of Toronto, Toronto, ON Canada; 131 Department of Neurology, University of Texas Southwestern, Dallas, TX USA; 132 Departments of Psychiatry, Medicine, Family & Community Medicine, South Texas Veterans Health Administration Geriatric Research Education & Clinical Center (GRECC), UT Health Science Center at San Antonio, San Antonio, TX USA; 133 Department of Radiology and Imaging Sciences, Indiana University, Indianapolis, IN USA; 134 Department of Pathology (Neuropathology), Rush University Medical Center, Chicago, IL USA; 135 Department of Psychiatry, University of Southern California, Los Angeles, CA USA; 136 Department of Population & Quantitative Health Sciences, Case Western Reserve University, Cleveland, OH USA; 137 Cambridge Institute for Medical Research, University of Cambridge, Cambridge, UK; 138 Department of Pathology, Johns Hopkins University, Baltimore, MD USA; 139 Sanders-Brown Center on Aging, Department of Neuroscience, University of Kentucky, Lexington, KY USA; 140 Department of Pathology & Laboratory Medicine, University of California Los Angeles, Los Angeles, CA USA; 141 Department of Psychiatry and Behavioral Sciences, Northwestern University Feinberg School of Medicine, Chicago, IL USA; 142 Department of Psychiatry & Behavioral Sciences, Duke University, Durham, NC USA; 143 Department of Genetics, University of North Carolina Chapel Hill, Chapel Hill, NC USA; 144 Department of Pathology, Oregon Health & Science University, Portland, OR USA; 145 Evelyn F. McKnight Brain Institute, Department of Neurology, Miller School of Medicine, University of Miami, Miami, FL USA; 146 Departments of Neurology, Pharmacology & Neuroscience, Texas Tech University Health Science Center, Lubbock, TX USA

**Keywords:** Genetic association study, Alzheimer's disease

## Abstract

Genetics play an important role in late-onset Alzheimer’s Disease (AD) etiology and dozens of genetic variants have been implicated in AD risk through large-scale GWAS meta-analyses. However, the precise mechanistic effects of most of these variants have yet to be determined. Deeply phenotyped cohort data can reveal physiological changes associated with genetic risk for AD across an age spectrum that may provide clues to the biology of the disease. We utilized over 2000 high-quality quantitative measurements obtained from blood of 2831 cognitively normal adult clients of a consumer-based scientific wellness company, each with CLIA-certified whole-genome sequencing data. Measurements included: clinical laboratory blood tests, targeted chip-based proteomics, and metabolomics. We performed a phenome-wide association study utilizing this diverse blood marker data and 25 known AD genetic variants and an AD-specific polygenic risk score (PGRS), adjusting for sex, age, vendor (for clinical labs), and the first four genetic principal components; sex-SNP interactions were also assessed. We observed statistically significant SNP-analyte associations for five genetic variants after correction for multiple testing (for SNPs in or near *NYAP1*, *ABCA7*, *INPP5D*, and *APOE*), with effects detectable from early adulthood. The *ABCA7* SNP and the *APOE2* and *APOE4* encoding alleles were associated with lipid variability, as seen in previous studies; in addition, six novel proteins were associated with the e2 allele. The most statistically significant finding was between the *NYAP1* variant and PILRA and PILRB protein levels, supporting previous functional genomic studies in the identification of a putative causal variant within the *PILRA* gene. We did not observe associations between the PGRS and any analyte. Sex modified the effects of four genetic variants, with multiple interrelated immune-modulating effects associated with the *PICALM* variant. In post-hoc analysis, sex-stratified GWAS results from an independent AD case–control meta-analysis supported sex-specific disease effects of the *PICALM* variant, highlighting the importance of sex as a biological variable. Known AD genetic variation influenced lipid metabolism and immune response systems in a population of non-AD individuals, with associations observed from early adulthood onward. Further research is needed to determine whether and how these effects are implicated in early-stage biological pathways to AD. These analyses aim to complement ongoing work on the functional interpretation of AD-associated genetic variants.

## Introduction

The rapidly decreasing cost of genomics paired with technological advances in the generation of multi-omic data has resulted in multiple datasets of deeply phenotyped individuals with a variety of health outcomes^1–3^. The data collected in these studies have the potential to yield important insights into potential molecular drivers of health observable in the blood periphery. The present study seeks to leverage a unique and relatively large set of multi-omic, deep-phenotyping data to shed light on genetic pathways to late-onset Alzheimer’s disease (AD) by assessing differences in ~ 2000 analytes in the blood that show association with known genetic risk variants for AD. Coupled with high-dimensional data sets, this approach has the potential to yield clues into gene pleiotropy, disease processes, and possible early-intervention strategies, which are critically important given the essentially untreatable nature of late-stage Alzheimer’s disease once significant brain deterioration has occurred.

Genetic variation plays a substantial role in AD risk, with twin studies estimating AD heritability at 58–79%^4^. While the emergence of recent large-scale consortia efforts has facilitated well-powered meta-analyses of genome-wide association studies (GWAS) to identify multiple common variants with small effect sizes^5,6^, the research community is still untangling exactly how this genetic variation influences disease risk. Functional genomics studies are beginning to identify likely genetic pathways to disease with the aid of transcriptomic, epigenomic, and endophenotypic data^7–10^. So far, genetic and multi-omic studies of AD studies have largely focused on older individuals with either clinically diagnosed AD or milder symptoms of cognitive decline, despite research pointing to highly variable AD pathobiology that occurs on a spectrum, and begins decades before clinical symptoms onset^11^.

In this study, we leveraged the results from a large-scale GWAS meta-analysis^5^ alongside data from a deeply phenotyped wellness cohort to investigate the physiological periphery effects of genetic risk for AD in individuals without known cognitive impairment, at all ages. We undertook an agnostic approach by adopting a phenome-wide association study (PheWAS) design^12^. By examining how genetic variation impacts 2008 analytes in the blood of 2831 individuals, we sought to complement previous functional genomics studies as well as potentially reveal new testable hypotheses for future studies. In addition, we tested for associations between a polygenic risk score (PGRS) for AD and blood analytes to determine if a relative burden of genetic risk might impact observable changes in the blood, and we assessed for effect modification of genetic risk by sex.

## Results

### Summary of population and study design

Sixty-one percent of Arivale participants were female, 22% were of non-white self-reported ethnicity, and 28% were obese (Table 1). The mean age at blood draw was 47 years, with a range of 18 to 89+. In general, individuals who joined Arivale had somewhat higher levels of cardiovascular risk markers compared to the US population, and slightly lower rates of obesity and pre-diabetes^3^ (these rates were consistent with rates in the geographies and ethnicities of the population, mostly from the west coast region of the United States).

**Table 1:** Baseline self-reported characteristics of Arivale participants with available whole-genome sequences.

Characteristic^a^	N = 2831
Age, mean (sd)	47.0 (12.0)
Female, n (%)	1719 (60.7)
**Nonwhite**^**b**^**, n (%) (n = 2725)**	597 (21.9)
Afro-Caribbean	1 (< 0.1)
American Indian or Alaska Native	5 (0.2)
Ashkenazi Jewish	49 (1.8)
Asian	84 (3.1)
Black or African American	64 (2.3)
East Asian	91 (3.3)
Hispanic Latino or Spanish origin	120 (4.4)
Middle Eastern or North African	18 (0.7)
Native Hawaiian or other Pacific Islander	17 (0.6)
Sephardic Jewish	4 (0.1)
South Asian	79 (2.9)
White	2128 (78.1)
Other	65 (2.4)
BMI, mean (sd) (n = 2750)	27.9 (6.4)
Obese^c^, n (%) (n = 2750)	802 (29.2)
Moderate activity ≥ 3×/week, n (%) (n = 2275)	1460 (64.2)
Vigorous activity ≥ 3×/week, n (%) (n = 2271)	697 (30.7)
Ever smoke, n (%) (n = 2207)	565 (25.6)
Current meds for cholesterol, n (%) (n = 2378)	287 (12.1)
**Past and/or current self-report of**	
Migraine, n (%) (n = 2229)	558 (25.0)
High cholesterol, n (%) (n = 2301)	558 (24.2)
Depression, n (%) (n = 2278)	521 (22.9)
GERD, n (%) (n = 2220)	464 (20.9)
Hypertension, n (%) (n = 2316)	434 (18.7)
Asthma, n (%) (n = 2361)	376 (15.9)

^a^For categories with missing data, total non-missing N is reported in parentheses.

^b^Race/ethnicity categories presented to participants in Arivale questionnaire.

^c^Obese defined as BMI ≥ 30.

### Phenome-wide association study results

**Figure 1:**
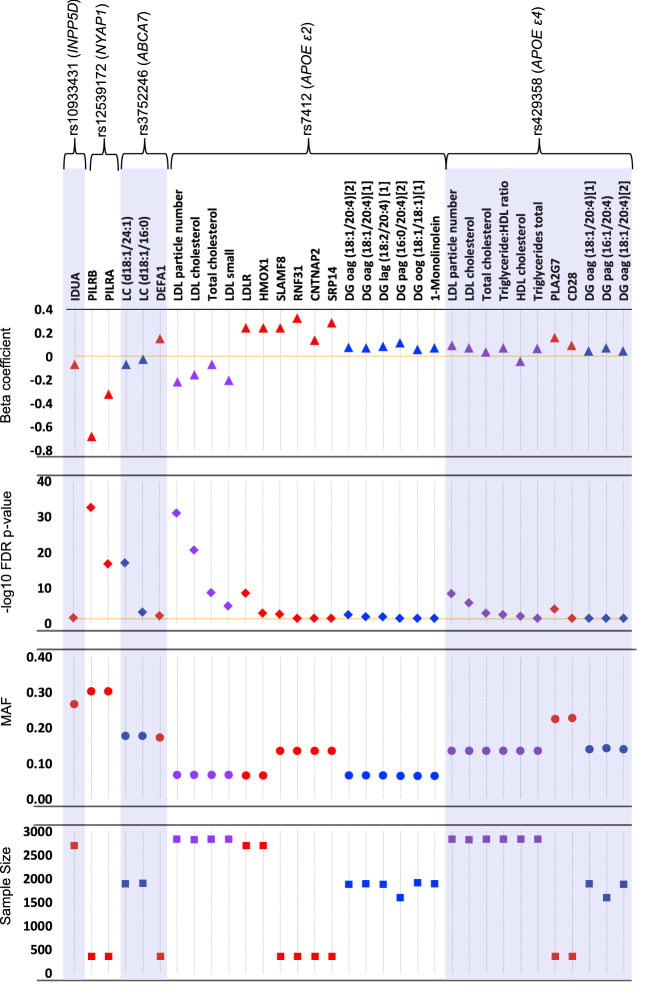
Statistically significant SNP-analyte associations after correcting for multiple testing (threshold FDR-adjusted p-value = 0.05), by SNP. Top panel: log-transformed beta-coefficient from the linear regression model adjusted for sex, age, and genetic principal components 1–4; markers above the zero line (orange) indicate analytes that increased in value with the minor allele, while markers below the line indicate markers that decreased in value. Second panel: FDR-adjusted − log_10_ p-value; orange line at FDR-p = 0.05. Proteins = red, metabolites = blue, clinical chemistries = purple. Metabolite codes: *DG* diacylglycerol, *LC* lactosylceramide, *o* oleoyl; *a* arachidonoyl, *g* glycerol, *l* linoleoyl, *p* palmitoyl. Third panel: minor allele frequency (MAF). Bottom panel: Total sample size for each analyte-SNP regression.

We observed 33 SNP-analyte associations that were statistically significant at FDR-adjusted p-value < 0.05, with most of the associations observed for the *APOE* SNPs (rs7412, or the e2-defining allele, and rs429358, or the e4-defining allele). The other SNPs showing significant associations with at least one clinical chemistry, protein, or metabolite were rs10933431, rs12539172, and rs3752246 (Fig. 1, Table S2). Complete PheWAS results, including beta coefficients, sample sizes, minor allele frequencies, Hardy–Weinberg Equilibrium p-values, and raw and adjusted p-values for each SNP are in Supplementary Excel File 1. Sample sizes varied among analytes collected (particularly among protein analytes, as a small subset of the population (N = 354) had samples submitted for the full range of protein panels, as described in “Methods” section).

#### NYAP1

**Figure 2:**
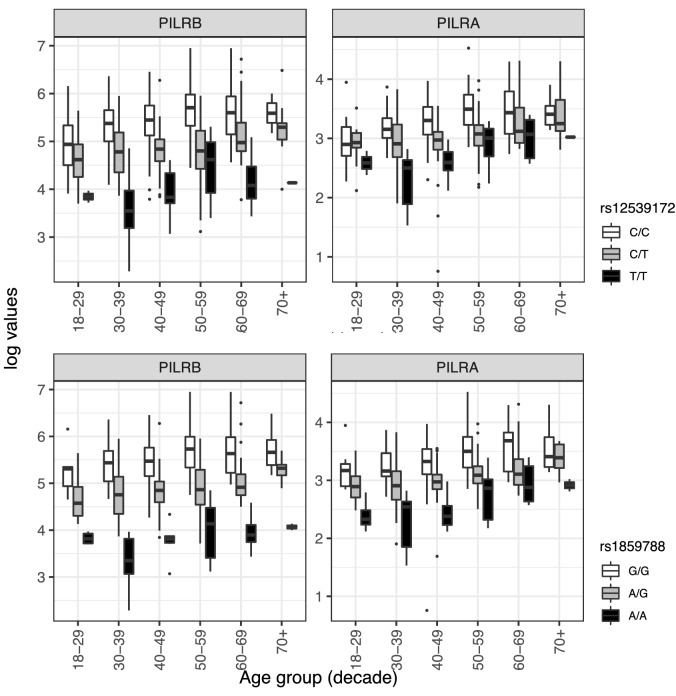
Unadjusted box plots of normalized protein expression (NPX) levels of PILRA and PILRB by genotype and age group. White boxplots = individuals who are homozygous for the major allele, gray boxplots = heterozygotes, black boxplots = minor allele homozygotes. Box plot midline = median value, lower/upper hinges = 25th and 75th percentiles, respectively; lower whisker ends/upper whisker ends no further than 1.5× interquartile range from the hinge. Data beyond whiskers are outlying points. Top panel: NPX of PILRA and PILRB by rs12539172 (NYAP1) genotype; Bottom panel: NPX of PILRA and PILRB by rs1859788 genotype.

The most robust SNP-analyte associations we observed were between rs12539712 in the 3’ region of *NYAP1* (Neuronal Tyrosine Phosphorylated Phosphoinositide-3-Kinase Adaptor 1), and two co-regulated proteins, paired immunoglobulin-like type 2 receptors beta and alpha (PILRB and PILRA) (Fig. 2). Carriage of the minor allele (AD risk odds ratio (OR) = 0.92) was associated with significant reduction in normalized protein expression (NPX) of PILRB and PILRA compared to individuals homozygous for the major allele (FDR-adjusted p-values = 2.2 × 10^–33^ and 2.3 × 10^–17^, respectively), while the overall level of NPX increased with age among all genotypes. The reduction in protein levels appears roughly dose-dependent with the number of minor alleles and was observed in all but the oldest and youngest age groups (likely due to small numbers of the minor allele in these groups (Table S3A). These observations led us to previous studies pointing to variation in *PILRA* as the causal gene at this locus, with a missense SNP as a leading candidate (G78R, rs1859788)^13–16^. In post-hoc analysis, we repeated the PheWAS with this putative causal SNP (which was in LD with our index SNP rs12539172, R^2^ = 0.77), and the associations became stronger (FDR-adjusted p-value for PILRB = 3.6 × 10^–52^; for PILRA = 1.4 × 10^–22^) (Fig. 2), with genotype differences observed in all age groups (Table S3A).

#### APOE4

We observed significant associations between rs429358 (which encodes the e4 allele) and multiple related clinical measures of cholesterol (Fig. S1). Differences by genotype were less pronounced in older age groups likely due to statin use (Table S3B); exploratory analyses visualizing only individuals who did not report use of statin-lowering medications showed more consistent genotype-dependent differences between rs429358 and the top cholesterol marker, low-density lipoprotein (LDL) particle number (Fig. S2, Table S3B). The concentration of two proteins in the blood were associated with the e4 allele: PLA2G7 (Platelet Activating Factor Acetylhydrolase) and CD28 (T-Cell-Specific Surface Glycoprotein CD28). Selected lipid metabolites in the blood were positively associated with e4: two diacylglycerol (DG) metabolites (one of which was measured twice in the Metabolon panel) were higher in e4 carriers compared to non-carriers.

#### APOE2

**Figure 3:**
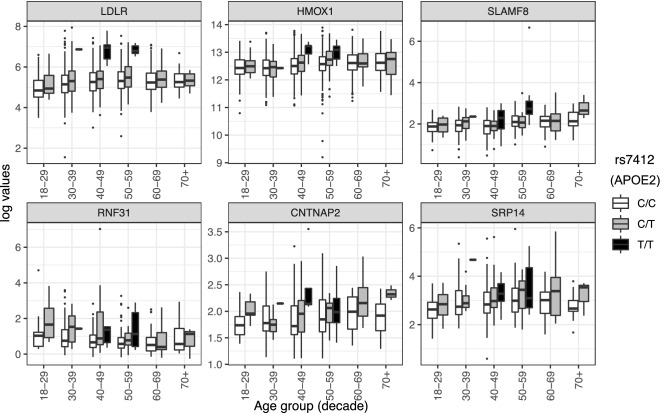
Unadjusted box plots of normalized protein expression levels (NPX) of six proteins significantly associated with APOE2 genotype, by age group. White boxplots = individuals who are homozygous for the major allele, gray boxplots = heterozygotes, black boxplots = minor allele homozygotes. Box plot midline = median value, lower/upper hinges = 25th and 75th percentiles, respectively; lower whisker ends/upper whisker ends no further than 1.5× interquartile range from the hinge. Data beyond whiskers are outlying points. *LDLR* low-density lipoprotein receptor, *HMOX1* heme oxygenase-1, *SLAMF8* SLAM family member 8, *RNF31* E3 ubiquitin-protein ligase RNF31, *CNTNAP2* contactin-associated protein-like 2, *SRP14* signal recognition particle 14 kDa protein.

We observed significantly lower levels of multiple clinical measures of LDL cholesterol associated with carriage of the e2 allele (Fig. S3). As the unadjusted plots show, e2 homozygotes are dramatically different than other genotypes, though it should be noted that few e2 homozygotes were present in the population (n = 16) and were within a limited age range (30–59 years). Selected lipid metabolites in the blood were positively associated with e2: a monoglyceride (MG) and four diacylglycerol (DG) metabolites (one of which was a replicate) were higher in e2 carriers compared to non-carriers. We observed six e2-protein associations (Fig. 3), such that each of the following proteins were observed at higher levels in e2 carriers: low density lipoprotein receptor (LDLR), heme oxygenase-1 (HMOX-1), SLAM family member 8 (SLAMF8), ring finger protein 31 (RNF31), contactin associated protein 2 (CNTNAP2), and signal recognition particle 14 (SRP14).

#### ABCA7

The *ABCA7* (ATP Binding Cassette Subfamily A Member 7) variant (rs3752246), which has been associated with increased risk of AD (OR 1.15, Table S1), was associated with lower levels of two lactosylceramide (LC) metabolites in the sphingolipid family. These differences were evident starting in the youngest age groups (Fig. S4, Table S3A). The minor allele of rs3752246 was also associated with higher levels of DEFA1 (Defensin Alpha 1), an antimicrobial peptide.

#### INPP5D

An intronic SNP in *INPP5D* (Inositol Polyphosphate-5-Phosphatase D) (rs10933431), which was associated with a lowered risk of AD in meta-analyses, was associated with lower levels of the protein IDUA (alpha-l-iduronidase) (Fig. S4).

#### Polygenic risk score

No associations were observed between the *APOE*-free PGRS and any analyte after FDR correction for multiple testing, either in primary analyses or in analyses adjusted for e4 status, or among non-e4 individuals only. No effect modification by sex or APOE4 status was observed.

### Sex-specific findings

**Figure 4:**
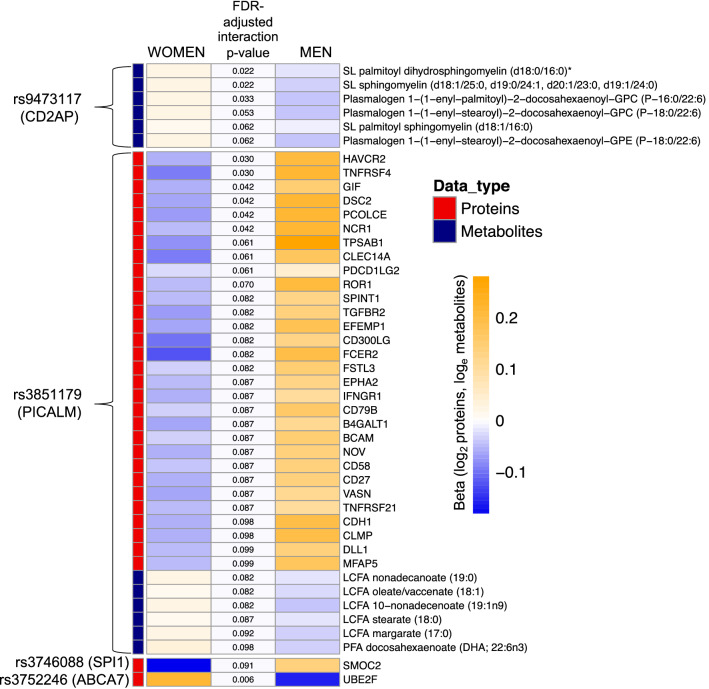
Heatmap of statistically significant genotype × sex interaction terms at FDR-adjusted p-value < 0.1. Beta coefficients obtained from sex-stratified analyses, middle-column p-values from interaction term in the full model. *SL* sphingolipid, *LCFA* long-chain fatty acid, *PFA* polyunsaturated fatty acid.

We observed a SNP × sex interaction involving the AD-protective *PICALM* variant, such that the minor allele was associated with higher levels of 30 proteins in men and lower levels of the proteins in women (Fig. 4, Fig. S5, Table S4). These proteins were highly correlated with one another (mean pairwise spearman’s rho = 0.49); thus, it is unclear whether the associations are independently biologically meaningful, or whether there is a passenger effect, in which one or a few proteins are driving the sex-differential association with genotype observed in the data. In addition, the *PICALM* variant is associated with a sex-specific effect on five highly correlated long-chain fatty acid (LCFA) metabolites and one polyunsaturated fatty acid (PFA) metabolite (Docosahexaenoic acid) (Fig. 4). To investigate further, we conducted a post-hoc analysis examining the impact of this variant on AD risk stratified by sex, in a meta-analysis of clinically diagnosed late-onset AD (18,812 individuals, Table S5). While AD risk was reduced in both men and women among carriers of the minor allele, the effect was stronger among men (Table 2, Table S6), which was consistent with the sex-stratified SNP-analyte analyses (data not shown).

**Table 2:** Results of sex-specific analysis and sex-SNP interaction analysis of PICALM variant rs3851179 in the ADGC.

Sex-stratified results^a^	Beta	Std error	p-value	MAF
Male model 1	− 0.206	0.035	5.62E−09	0.358
Male model 2	− 0.176	0.038	4.08E−06	0.359
Female model 1	− 0.083	0.029	4.37E−03	0.354
Female model 2	− 0.087	0.031	5.60E−03	0.352

Interaction results^b^	Interaction beta	Std error	p-value	MAF
Model 1	0.116	0.044	8.05E−03	0.354
Model 2	0.372	0.048	7.84E−02	0.354

N = 9135 cases (60% female), 9,677 controls (60% female).

^a^Model 1: adjusted for age, sex, and PCs; Model 2: adjusted for age, sex, PCs, and APOE genotype.

^b^Model 1: adjusted for age and PCs; Model 2: adjusted for age, PCs, and APOE.

Other observed sex-specific effects were more modest. The SNP near *CD2AP* (CD2 Associated Protein) interacted with sex to affect three highly correlated sphingomyelins and three plasmologens, while the SNP in *SPI1* (Transcription Factor PU.1) interacted with sex to affect SPARC related modular calcium binding 2 (SMOC2). Lastly, the missense *ABCA7* SNP interacted with sex to affect levels of Ubiquitin conjugating enzyme E2f (UBE2F).

### Stratification by self-identified race/ethnicity

Unfortunately, due to vanishingly small numbers in individual self-identified groups (Table 1), we were not able to assess genetic risk effects in individual groups with statistical rigor. As expected, analyses restricted to white individuals recapitulated results of the overall analysis (Fig. S6). In the nonwhite group overall, we observed effect sizes that were consistent with the overall results and white-only results (Fig. S7).

## Discussion

Our study examines associations between known genetic risk factors for AD and blood markers (clinical labs, proteins, and metabolites). It provides insight into the manifestation of AD-related genetic risk in blood-borne analytes from cognitively normal individuals and demonstrated how AD-related genetic variation manifests in the blood across adulthood. Our results contribute to the growing literature highlighting a potential causal variant (missense SNP in *PILRA),* point to potential new mechanisms of protection among *APOE2* carriers, and suggest a role for infectious diseases as AD risk factors, alongside lipid metabolism, immune response, and endocytosis. We also uncovered intriguing differences between men and women in how genetic risk manifests in the blood. These analyses not only add to the existing literature on functional genomics in AD, but also offer up multiple potential new hypotheses to catalyze future studies.

The strongest associations in the study were between the *NYAP1* SNP (rs12539172) and the PILRB/PILRA proteins. This locus was originally identified by rs1476679 near *ZCWCP1*^6^. *NYAP1* and *ZCWPW1* are located near *PILRA* and *PILRB* on chromosome 7, within a linkage disequilibrium (LD) block. In previous gene expression studies, the initial index SNP for *ZWCWP1* has been associated with expression of multiple *PILRB* and *PILRA* transcripts in brain^9,17^. *PILRA* and *PILRB* are paired, co-regulated inhibiting/activating receptors, respectively, that are expressed on innate immune cells, recognize certain O-glycosylated proteins, and have an important role in regulating acute inflammatory reactions^18^. The R78 substitution in *PILRA* (rs1859788) has been shown to reduce the binding capacity of endogenous ligands and thereby potentially increase microglial activity^16^. In addition, while controversial, work from our group and others^19–21^ has suggested a potential viral role in AD risk. Notably, the R78 variant has been implicated in HSV-1 (Herpes Simplex Virus type 1) infectivity^16^ and differences in HSV-1 antibody titer levels^13^. While previous studies have hypothesized that reduced activity of *PILRA* was due to steric conformational changes in the protein leading to reduced binding of key ligands (including HSV-1 glycoprotein B), our results suggest that reduced levels of circulating PILRA protein in R78 carriers could also be a factor in the overall protective effect of this genetic variant.

Statistically significant associations were observed between multiple lipid analytes and the SNPs encoding both *APOE4* (rs429358) and *APOE2* (rs7412). *APOE* normally plays a key role in lipid transport, including shuttling cholesterol to neurons in healthy brains. Notably, *APOE* has a role in beta-amyloid (Aβ) metabolism, and while the exact mechanism is unknown, the e4 variant appears to accelerate neurotoxic Aβ accumulation, aggregation, and deposition in the brain^22^. The associations we observed between the e4 variant and increased levels of total cholesterol and LDL cholesterol, along with lower levels of high-density lipoprotein (HDL), were consistent with previous cardiovascular disease cohort studies that included young, middle-aged, and older adults^23–26^. The e4 allele was associated with increased NPX of two inflammatory proteins. PLA2G7 is a known cardiovascular risk marker with pro-inflammatory and oxidative activities^27^ which has previously been associated with *APOE* genotypes^28^ and implicated in AD and cognitive decline^27,29^. To our knowledge, CD28 protein levels have not previously been associated with e4 status, though this relatively weak association may be a downstream result of *APOE* isoform-specific effects on inflammation^30^.

Blood cholesterol levels among *APOE2* carriers were also largely consistent with a body of existing data^24^; the e2 variant was associated with lower levels of multiple measures of LDL cholesterol. It should be noted that while 5–10% of e2 homozygotes develop type III hyperlipoproteinemia (typically in the presence of an existing metabolic disorder^31^) resulting in elevated cholesterol levels, all e2 homozygotes in the study had significantly decreased levels of LDL cholesterol compared to other genotypes. In contrast, the e2 variant was associated with higher levels of six lipid metabolites in the diacylglycerol and monoacylglycerol family; interestingly, both the e4 variant and e2 variants were associated with increased levels of the same two lipid metabolites in the diacylglycerol family, despite the opposite effects of these two variants on circulating blood cholesterol. Diacylglycerol is a precursor to triacylglyceride (TG), which is typically higher in *APOE2* carriers^26^. The effects of high DGs and TGs remains unclear. DG-rich diets fed to diabetic *APOE*-knockout mice had reduced atherosclerosis and lower plasma cholesterol than mice fed TG-rich or western diets^32,33^; however, non-targeted metabolomics studies have shown elevated levels of DGs and MGs in AD and mild cognitive impairment (MCI) patient brains and blood compared to cognitively intact individuals^34,35^.

We observed six proteins that were significantly upregulated in *APOE2* carriers (Fig. 3). The LDLR protein had higher levels of NPX in e2 carriers, particularly in e2 homozygotes. Though *APOE2* is known to bind poorly to LDLR (~ 2% of e3 or e4 binding activity)^36^, *APOE2* was associated with lower levels of LDL cholesterol across age groups as noted previously, perhaps due to compensatory up-regulation of *LDLR*^26^. Greater understanding of the compensatory mechanism leading to upregulated *LDLR* and lower circulating LDL cholesterol is needed. The e2 variant was associated with increased levels of the highly inducible *HMOX-1*, which has antioxidant properties and has been associated with both neuroprotection and neurodegeneration^37^. *SLAMF8* may be another link to an antioxidant effect of *APOE2,* as it has been implicated in modulation of reactive oxygen species and inflammation via negative regulation of NOX activity^38^. *APOE2* carriers displayed higher levels of RNF31 protein (aka *HOIP*). *HOIP* is the catalytic component of the linear ubiquitin chain assembly complex (LUBAC), which was shown to have a role in the recognition and degradation of misfolded proteins^39^. Variation in *CNTNAP2*, a member of the neurexin superfamily of proteins involved in cell–cell interactions in the nervous system, has been associated with neurodevelopmental disorders^40^, and has been implicated in AD-related dementia^41^. Lastly, *SRP14*, which has a role in targeting secretory proteins to the rough endoplasmic reticulum (ER) membrane, has been identified as one of many tau-associated ER proteins in AD brains^42^. To our knowledge, the *APOE2*-protein associations described here are novel and may help point to the mechanisms of protection associated with the e2 variant.

*ABCA7* is involved in lipid efflux from cells into lipoprotein particles, plays a role in lipid homeostasis^43^, and has also been implicated in Aβ processing and deposition in the brain^44^. Our results support *ABCA7*’s lipid-related function by showing lower levels of two LC metabolites among individuals carrying the AD-risk allele of rs3752246. In contrast, we observed higher NPX of DEFA1 protein in carriers of the *ABCA7* variant, which is consistent with previous studies showing higher levels of this protein in cerebral spinal fluid (CSF) and sera of AD patients compared to controls^45,46^, potentially linking *ABCA7* with an inflammatory response pathway to AD. Lastly, lower NPX of IDUA was associated with the *INPP5D* SNP. *INPP5D*, which encodes the lipid phosphatase SHIP1, is a negative regulator of immune signaling and is expressed in microglia^47^. To our knowledge, this association has not been previously observed.

Genetic variation likely affects men and women differentially, pointing to mechanisms that contribute to known differences in AD pathology between the sexes^48^. The set of proteins that were differentially affected by sex and *PICALM* genotype are primarily implicated in immune processes, cell adhesion, and regulatory processes, with widely overlapping functions (Fig. S8). Our results highlight an interaction between the AD-risk variant in *PICALM* and multiple proteins implicated in immune response in a sex-specific manner, and support emerging research showing sex differences in the neuroimmune response that impact microglia function^49^. We also observed a sex-differential effect of the variant on multiple LCFA metabolites and one PFA metabolite (DHA). A potential link between *PICALM* function, lipids, and AD is feasible: fatty acids, and DHA in particular, have long been known to have a role in maintaining brain health and cognition^50^, while *PICALM* expression has been shown to influence cholesterol homeostasis through multiple mechanisms^51^. This multi-analyte interaction was supported by results from sex stratified GWAS meta-analyses, which showed differing effect sizes of the variant on men vs. women.

In addition to these sex-specific *PICALM* effects, the SNP near *CD2AP*, a scaffolding protein, interacted with sex to affect three highly correlated sphingomyelins and three plasmologens, while the SNP in *SPI1*, a transcription factor associated with microglial activation^52^, interacted with sex to affect SMOC2, a protein involved in microgliosis that has been previously associated with Aβ positivity in CSF^53^.

We also examined an AD-specific polygenic risk score. While the PGRS is predictive of disease in case/control studies^54^, it was not associated with any blood analytes in the all-ages AD-free Arivale cohort. Combining genetic effects into a single score for AD likely served to dilute any individual genetic effect on the manifestation of genetic risk in the blood. In addition, the relative youth and cognitive health of this cohort should be considered. The PGRS may be more likely to detect perturbation in analytes that are markers of systemic inflammation or immune dysfunction in later life and among cohorts experiencing cognitive impairment.

The results presented here are novel and we believe will be of interest to the AD-related functional genomics community, though several limitations should be noted. The study population was not a random sample but was self-selected. The population is largely self-identified non-hispanic white, was mostly located on the west coast, and likely has higher than average socio-economic status (though these data were not captured). Thus, results may not be generalizable to a broader population. At this time, we were not aware of a suitable replication cohort that would contain parallel-omics panels in an all-ages health-heterogeneous cohort. Future studies will be needed to assess generality of the findings to other populations, not only for the sake of replicability of the findings, but due to the relative ancestral homogeneity of this data set. Previous studies have shown genetic heterogeneity between white and non-white individuals, particularly with regard to African Americans and risk of cognitive outcomes among carriers of *APOE* and *ABCA7* variants^55,56^. Given known wide-ranging racial/ethnic disparities in dementia incidence^57^, it is imperative that future deep-phenotyping studies are far more inclusive than the study presented here.

Another limitation to the interpretation of results concerns the issue of pleiotropy; we cannot discern pleiotropic, non-AD-related effects from true causal effects that are implicated in AD pathogenesis. However, even if the associations described here are purely the result of pleiotropy and are unrelated to causal mechanisms of AD, the novel associations we described may provide clues to the function of several genes that are highly interesting to the AD community. Related, we only obtained peripheral plasma, and are unable to examine effects in AD-relevant compartments such as brain or CSF. We had high-coverage WGS available and did not interrogate other types of genetic variation such as copy number variants, indels, and short tandem repeats. Lastly, data harmonization with other studies will be a challenge. For instance, most previous metabolomics studies used metabolomics data that lacked complete speciation, and more work is needed with newer technologies that yield high fidelity data to determine the biological effects of specific serum metabolites.

This study also has multiple strengths. While most studies focused on AD-related genetic variation consist of case/control cohorts in older adults, the Arivale data offered an unprecedented look into how genetic variation perturbs physiological pathways in the blood long before disease onset, in health-heterogeneous individuals of all ages. This feature allowed us to observe subtle changes in blood associated with genetic variation, due to the relatively large sample size (2831 individuals with WGS) and the high quality of the blood analytes collected. Our results are from a “real-world” cohort, which promises to be an increasing source of large-scale data in the community going forward, with its accompanying advantages and disadvantages. Some results were previously unobserved and need to be replicated (such as the associations between *APOE2* and multiple proteins), while other results agree with previous findings and serve to reinforce confidence that the results are reasonably representative and not simply spurious.

## Conclusions

Due to a unified world-wide effort, dozens of genetic variants have been robustly implicated in the development of AD, though we are still in the early stages of understanding exactly how genetic variation contributes to disease. Our study showed that AD-related genetic variation manifests in the blood, from early adulthood onward, and highlights known targets for prevention in early and mid-life, such as cholesterol monitoring, mitigation of inflammation, and possibly, HSV-1 prevention and/or viral load management. Importantly, as well as yielding new insight into the pathobiology of AD through adulthood, these results may provide a significant number of new drug targets that are highly novel and biologically plausible or may serve as biomarkers if confirmed to have a consistent influence on AD pathophysiology. Lastly, these results highlight the need to assess for sex differences in future studies. Taken together, these results not only illustrate previously unobserved biological phenomenon as a result of AD-associated genetic variation, but also serve as an important resource for the generation of hypotheses for future functional genomics studies and emphasize the potential insight that can be gleaned from deeply phenotyped individuals.

## Methods

### Population

The Institute for Systems Biology, through partnership with their spin-out company Arivale, has access to a wealth of data collected from subscribers in the commercially available (now closed) Arivale Scientific Wellness program^3,58^, from July 2015 to May 2019. In brief, participants in the Arivale program were assigned a health coach upon joining the program, who then utilized data from clinical blood assays and detailed health-history and behavioral questionnaires to personalize health advice and management of health goals.

All research was conducted in accordance with regulations and guidelines for observational research in human subjects. Informed consent was obtained from all participants for the use of their anonymized data in research. The study was reviewed and approved by the Western International Review Board (Study Number 1178906 at Arivale and Study Number 20170658 at the Institute for Systems Biology, in Seattle, WA).

### Blood-derived clinical laboratory tests and whole genome sequencing

We identified 2831 individuals with whole genome sequencing (WGS) and at least one class of blood-derived analyte, described as follows. For each participant, fasting clinical blood laboratory tests were measured upon joining the program. Blood samples were collected at either local facilities hosted by LabCorp (North Carolina, USA) or Quest Diagnostics (New Jersey, USA). Whole genome sequencing was performed on DNA extracted from whole blood with library preparation using the Illumina TruSeq Nano Library prep kit and sequenced using Illumina HiSeq X, PE-150, target 30× coverage at a single Clinical Laboratory Improvement Amendmnets (CLIA)-approved sequencing laboratory. Only values with < 20% missing were included, and no imputation was performed. At the baseline blood draw, 2827 of the 2831 individuals with sequenced whole genomes had up to 63 fasting clinical blood lab tests. Clinical blood tests included standard markers for cardiometabolic health (lipid levels), diabetes, inflammation, kidney and liver function, nutrition (vitamins and minerals), and blood cell counts. All clinical lab tests included, with descriptions and units where available, are in Supplementary Excel File 2.

Proteomics: Frozen plasma samples (aliquots of the initial blood draw) were also sent to Olink (Olink Bioscience, Sweden) for targeted proteomics assays based on Olink’s proximity extension assay (PEA) technique^59^, which is a dual-recognition, DNA-coupled methodology that is quantified by quantitative real-time PCR and enables high multiplex, high throughput proteomics that are both sensitive and specific (for further details, see https://www.olink.com/our-platform/our-pea-technology/). Full details of normalization and batch effect adjustment have been described previously^60^. For analysis, only proteins with < 20% missing were included and no imputation was performed. Up to 2694 of these participants had quantitative proteomic data on 274 proteins from three Olink panels (Cardiovascular II, Cardiovascular III, and Inflammation panels). An additional 919 proteins (from 10 additional panels available at Olink at the time) were obtained from a subsample of 354 individuals, in which *Apolipoprotein E* (*APOE*) e2/e2 and *APOE* e4/e4 genotypes were overrepresented. Since multiple batches were performed, previously generated pooled control samples were run with each batch and used for batch correction and multiple control samples were included on each plate.

#### Metabolomics

Aliquots of frozen plasma samples were sent to Metabolon, Inc. (North Carolina) to conduct metabolomics assays using the Metabolon HD4 discovery platform. In brief, Metabolon conducted their Global Metabolomics high-performance liquid chromatography (HPLC)-mass spectrometry assays on the plasma samples. Full details of sample handling, quality control, biochemical identification, data curation, and quantification and normalization has been described previously^60,61^. For analysis, only metabolites with < 20% missing (or detectable) were included and no imputation was performed. Up to 1909 of the participants had data from 754 metabolites, though due to technical variability and variation in detection rates of rare metabolites, sample sizes ranged from 1539 to 1909 after pruning metabolites with < 20% missing. Relative concentration values were reported for each metabolite. Full biochemical annotation for each metabolite (when available), as provided by Metabolon at the time of quantification, can be found in Supplementary Excel File 2.

### SNP selection

We selected 25 common and somewhat-rare (> 1% allele frequency) single nucleotide polymorphisms (SNPs) that were significantly associated with AD in a large-scale meta-analysis based on updated data from the International Genomics of Alzheimer’s Project (IGAP)^5^. In addition to these variants, we also included the SNP coding for *APOE* e2 (rs7412). The 25 SNPs were linked to 24 genes (two SNPs in *APOE*), as detailed in Table S1.

### Polygenic risk score calculation for AD

PGRS for age-associated AD risk was computed using summary statistics from the initial IGAP-driven GWAS meta-analysis^6^. Briefly, the set of SNPs included in the PGS was determined as follows. The Benjamini–Hochberg^62^ procedure was applied to the p-values for all SNPs tested in the GWAS to account for multiple testing by controlling the false discovery rate (FDR) at a 5% level. This FDR-filtered set of SNPs was then further pruned using linkage disequilibrium (LD): pairs of SNPs in close proximity capturing highly correlated information (r^2^ > 0.2) were identified, and the SNP with the smaller p-value in the pair was kept; this was repeated until all remaining SNPs were mutually uncorrelated (r^2^ < 0.2 for all pairs). The PGRS for each individual was then calculated by summing up the published effect size for each selected SNP multiplied by the number of effect alleles the individual carried for that SNP, across all of the selected SNPs. Missing genotypes were mean imputed using the effect allele frequency.

### Statistical analysis

Following a phenome-wide association study approach (PheWAS)^12,63^, the primary model for each SNP used linear regression, with genotype (0, 1, or 2, with 0 indicating homozygosity for the major allele and 2 indicating homozygosity for the minor allele) as the predictor, and each continuous quantitative analyte as the dependent variable. Clinical lab and metabolite values were natural log transformed to account for right skewness and outliers, with + 1 added to each natural log transformation to prevent zero values. Proteomic quantities were presented as normalized protein expression (NPX), Olink’s arbitrary unit, which is in log2 scale. Genetic ancestry was represented by principal components (PCs) 1–4, calculated using previously described methods^64^. All SNP models were adjusted for age, sex, genetic ancestry PCs 1–4, and vendor identification for the clinical labs. Secondary models tested effect modification by sex by including a gene x sex interaction term in the models. We accounted for multiple comparisons by applying the Benjamini–Hochberg method^62^ at alpha = 0.05 on a per-SNP basis and applied to the main effect of genotype in the primary models, while we set B-H alpha = 0.1 of the sex-SNP interaction term as the threshold for the gene x sex interaction models. The FDR rate took into account testing for all 2008 possible analytes, with the understanding that this adjustment was highly conservative given a high degree of correlation among multiple groups of analytes, and the fact that some analytes were sampled in only a subset of individuals. Both raw and adjusted p-values are reported.

We also repeated the primary PheWAS approach with participants stratified by self-identified race, due to evidence for variable genetic risk for cognitive outcomes between non-Hispanic white (hereafter referred to as “white”) and non-white populations^55,56^. Unfortunately, due to small numbers of individuals in specific non-white racial and ethnic groups, which become vanishingly small when accounting for allele frequency and numbers of available samples (Table 1), we were not able to assess genetic risk effects in individual groups with statistical rigor and had to group all non-white participants into one stratum for analysis. The stratified white and non-white group analyses serve as an investigation into whether our primary results reflected the majority-white makeup of the Arivale population. PheWAS was applied as described above, with FDR to account for multiple comparisons.

To visualize genotype-analyte associations across adulthood, we created boxplots of the log-transformed analyte values by genotype, stratified by age group (by decade, from 18–29 to 70 and over). One-way analysis of variance (ANOVA) was used to test whether there was an overall difference between genotypes within each age group. All statistical analyses were performed in R v3.5.1 (https://www.R-project.org/).

In post-hoc exploratory analysis focused on the SNP in the *PICALM* (Phosphatidylinositol Binding Clathrin Assembly Protein) locus (rs3851179), sex-stratified and sex-interaction analyses was performed on 12,324 cases (57.7% female) and 11,453 controls (59.9% female) of European ancestry from the Alzheimer’s Disease Genetics Consortium (ADGC) (see Supplementary Table 4 for dataset details). Datasets were imputed to the Haplotype Reference Consortium (HRC)^65^ panel using the Michigan Imputation Server (https://imputationserver.sph.umich.edu/index.html#!). Standard pre-imputation quality control was performed on all datasets individually, including exclusion of individuals with low call rate, individuals with a high degree of relatedness, and variants with low call rate^66^. Individuals with non-European ancestry according to principal components analysis of ancestry-informative markers were excluded from the further analysis. Detailed descriptions of individual ADGC datasets can be found in Kunkle et al.^5^ and Table S5. Study-specific logistic regression analyses employed Plink^67^ for sex-interaction analysis and SNPTest^68^ for sex-stratified analysis. Sex-interaction, which analyzed the sex × variant interaction, and sex-stratified analysis of males and females separately, were performed for two separate models per analysis, one adjusting for age, sex and PCs (model 1) and a second adjusting for age, sex, PCs and *APOE* (model 2). Results were meta-analyzed with METAL using inverse variance-based analysis^69^. In order to explore the relationships among the proteins associated with the PICALM variant, we input the list of sex-interacting proteins into Cytoscape software, utilizing the CLUEGO plug-in^70,71^, which drew a network linking proteins through their known GO Biological processes.

## Supplementary Information

Supplementary Information 1.

Supplementary Information 2.

Supplementary Information 3.

## Data Availability

The datasets generated and/or analysed during the current study are not publicly available because the data was generated by a private investment firm under legal terms that mandate researchers to sign a data access agreement permitting the use of these data for non-proift research purposes. Upon reasonable request, researchers can access the Arivale deidentified dataset supporting the findings in this study for research purposes from ISB. Requests should be sent to data-access@isbscience.org. The data are available to qualified researchers on submission and approval of a research plan.

## Code availability

Code used for PheWAS statistical analysis is available through the Sage Bionetworks Github (https://github.com/Sage-Bionetworks/ADsnps_PheWAS.git).
