# Supplementary material for: Manifestations of Alzheimer’s disease genetic risk in the blood are evident in a multiomic analysis in healthy adults aged 18 to 90
Source: Sci Rep. 2022 Apr 12;12:6117. doi: 10.1038/s41598-022-09825-2 (PMC9005657; doi:10.1038/s41598-022-09825-2)

Supplementary Table 1. Replicated candidate SNPs associated with AD in Kunkle et al., 2019, GWAS meta-analysis

SNP	Variant class	Nearest gene	Chromosome	MAF[a]	Major/ Minor Allele	OR[b]	95% CI[b]	Meta-P[b]
rs4844610	intronic	CRI1	1	0.16	C/A	1.17	1.13-1.21	3.6e-24
rs6733839	intronic	BIN1	2	0.39	C/T	1.20	1.17-1.23	2.1e-44
rs10933431	intronic	INPP5D	2	0.27	C/G	0.91	0.88-0.94	3.4e-09
rs9271058	intergenic	HLA-DRB1	6	0.27	T/A	1.10	1.07-1.13	1.4e-11
rs9473117	intergenic	CD2AP	6	0.26	A/C	1.09	1.06-1.12	1.2e-10
rs12539172	3' UTR	NYAP1	7	0.30	C/T	0.92	0.90-0.95	9.3e-10
rs10808026	intronic	EPHA1	7	0.19	C/A	0.90	0.88-0.93	1.3e-10
rs73223431	intronic	PTK2B	8	0.35	C/T	1.10	1.07-1.13	6.3e-14
rs9331896	intronic	CLU	8	0.39	T/C	0.88	0.85-0.90	4.6e-24
rs7920721	upstream	ECHDC3	10	0.35	A/G	1.08	1.06-1.11	1.8e-11
rs3740688	intronic	SPI1	11	0.45	T/G	0.92	0.89-0.94	5.4e-13
rs7933202	intergenic	MS4A2	11	0.37	A/C	0.89	0.87-0.92	1.9e-19
rs3851179	intergenic	PICALM	11	0.36	C/T	0.88	0.86-0.90	6.0e-25
rs11218343	intronic	SORL1	11	0.06	T/C	0.80	0.75-0.85	2.9e-12
rs17125924	intronic	FERMT2	14	0.09	A/G	1.14	1.09-1.18	1.4e-09
rs12881735	intronic	SLC24A4	14	0.21	T/C	0.92	0.89-0.94	7.4e-09
rs593742	intergenic	ADAM10	15	0.36	A/G	0.93	0.91-0.95	6.8e-09
rs7185636	intronic	IQCK	16	0.20	T/C	0.92	0.89-0.95	2.4e-08
rs62039712	intergenic	WWOX	16	0.10	G/A	1.16	1.10-1.23	3.7e-08
rs138190086	intergenic	ACE	17	0.02	G/A	1.30	1.19-1.42	5.3e-09
rs3752246	missense	ABCA7	19	0.18	C/G	1.15	1.11-1.18	3.1e-16
rs7412	missense	APOE (ε2 allele)	19	0.07	C/T	*NA*		
rs429358	missense	APOE (ε4 allele)	19	0.13	T/C	3.32	(3.20-3.45)	1.2e-881
rs6024870	intronic	CASS4	20	0.08	G/A	0.88	0.85-0.92	3.5e-08
rs2830500	intergenic	ADAMTS1	21	0.29	C/A	0.93	0.91-0.96	2.6e-08

[a]MAF in Arivale cohort

[b]Kunkle et al Meta-analysis results, except rs429358 (stage 1 results posted) and rs7412, which was not included in the previous study.

Supplementary Table 2. AD-GWAS SNPs significantly associated with analytes in Arivale data (FDR p<0.05)

SNP	Analyte	Beta	SE	Un-adjusted p	FDR-adjusted p	Minor allele freq-uency	Sample N
SNPs associated with clinical chemistries							
rs429358 / APOE (e4)	LDL particle number	0.094	0.013	2.84E-12	5.71E-09	0.13	2824
rs429358 / APOE (e4)	LDL cholesterol	0.071	0.012	2.03E-09	2.04E-06	0.13	2817
rs429358 / APOE (e4)	Total cholesterol	0.034	0.007	3.61-E06	1.81E-03	0.14	2825
rs429358 / APOE (e4)	Triglyceride:HDL ratio	0.071	0.016	9.67E-06	3.88E-03	0.14	2825
rs429358 / APOE (e4)	HDL cholesterol	-0.044	0.010	2.25E-05	1.09E-02	0.14	2825
rs429358 / APOE (e4)	Triglycerides	0.065	0.017	1.41E-04	3.96E-02	0.14	2825
rs7412 / APOE (e2)	LDL particle number	-0.221	0.018	5.19E-35	1.04E-31	0.07	2824
rs7412 / APOE (e2)	LDL cholesterol	-0.160	0.016	2.95E-24	2.96E-21	0.07	2817
rs7412 / APOE (e2)	Total cholesterol	-0.068	0.010	3.88E-12	2.60E-09	0.07	2825
rs7412 / APOE (e2)	LDL small	-0.205	0.035	4.43E-08	1.78E-05	0.07	2824
SNPs associated with proteins							
rs10933431 / INPP5D	IDUA (P35475)	-0.072	0.017	1.51E-05	3.02E-02	0.27	2691
rs12539172 / NYAP1	PILRB (Q9UKJ0)	-0.683	0.048	1.12E-36	2.24E-33	0.31	354
rs12539172 / NYAP1	PILRA (Q9UKJ1)	-0.326	0.033	2.29E-20	2.30E-17	0.31	354
rs3752246 / ABCA7	DEFA1 (P59665)	0.149	0.034	1.24E-05	8.28E-03	0.17	353
rs429358 / APOE (e4)	PLA2G7 (Q13093)	0.159	0.030	1.94E-07	1.30E-04	0.23	353
rs429358 / APOE (e4)	CD28 (P10747)	0.091	0.024	1.41E-04	3.96E-02	0.23	354
rs7412 / APOE (e2)	LDLR (P01130)	0.240	0.035	7.39E-12	3.71E-09	0.07	2692
rs7412 / APOE (e2)	HMOX1 (P09601)	0.120	0.026	5.89E-06	1.69E-03	0.07	2691
rs7412 / APOE (e2)	SLAMF8 (Q9P0V8)	0.240	0.055	1.47E-05	2.80E-03	0.14	354
rs7412 / APOE (e2)	RNF31 (Q96EP0)	0.326	0.088	2.58E-04	4.28E-02	0.14	354
rs7412 / APOE (e2)	CNTNAP2 (Q9UHC6)	0.136	0.037	3.36E-04	4.43E-02	0.14	354
rs7412 / APOE (e2)	SRP14 (P37108)	0.287	0.079	3.53E-04	4.43E-02	0.14	354
SNPs associated with metabolites							
rs3752246 / ABCA7	Sphingolipid, N-nervonoyl-sphingosine (d18:1/24:1)*	-0.070	0.007	5.98E-21	1.20E-17	0.18	1884
rs3752246 / ABCA7	Sphingolipid, N-palmitoyl-sphingosine (d18:1/16:0)	-0.025	0.005	9.40E-07	9.43E-04	0.18	1895
rs429358 / APOE (e4)	oleoyl-arachidonoyl-glycerol (18:1/20:4) [1]*	0.043	0.012	1.90E-04	3.96E-02	0.14	1883
rs429358 / APOE (e4)	palmitoyl-arachidonoyl-glycerol (16:1/20:4)	0.069	0.019	2.01E-04	3.96E-02	0.14	1589
rs429358 / APOE (e4)	oleoyl-arachidonoyl-glycerol (18:1/20:4) [2]*	0.044	0.012	2.17E-04	3.96E-02	0.14	1873
rs7412 / APOE (e2)	oleoyl-arachidonoyl-glycerol (18:1/20:4) [2]*	0.075	0.016	4.80E-06	1.61E-03	0.07	1873
rs7412 / APOE (e2)	oleoyl-arachidonoyl-glycerol (18:1/20:4) [1]*	0.072	0.016	9.26E-06	2.32E-03	0.06	1883
rs7412 / APOE (e2)	linoleoyl-arachidonoyl-glycerol (18:2/20:4) [1]*	0.083	0.019	1.34E-05	2.80E-03	0.06	1872
rs7412 / APOE (e2)	palmitoyl-arachidonoyl-glycerol (16:0/20:4) [2]*	0.113	0.026	1.53E-05	2.80E-03	0.06	1593
rs7412 / APOE (e2)	oleoyl-oleoyl-glycerol (18:1/18:1) [1]*	0.057	0.016	2.78E-04	4.28E-02	0.06	1903
rs7412 / APOE (e2)	Monoacylglycerol, 1-linoleoylglycerol (18:2)	0.071	0.020	2.99E-04	4.28E-02	0.06	1888

Supplementary Table 3A. SNP-analyte ANOVA p-values by age group

SNP	Analyte	Age Group	ANOVA p-value[a]	SNP	Analyte	Age Group	ANOVA p-value[a]
rs12539712	PILRB	18-29	*9.15E-02*	rs7412	SLAMF8	18-29	*6.00E-01*
		30-39	**3.65E-10**			30-39	*3.03E-01*
		40-49	**3.60E-09**			40-49	*4.60E-01*
		50-59	**6.38E-12**			50-59	**2.18E-05**
		60-69	**2.10E-04**			60-69	*3.86E-01*
		70+	*2.62E-02*			70+	*7.24E-02*
rs12539712	PILRA	18-29	*5.24E-01*	rs7412	RNF31	18-29	*2.14E-01*
		30-39	**9.15E-07**			30-39	*3.92E-01*
		40-49	**4.06E-06**			40-49	**1.39E-03**
		50-59	**2.13E-08**			50-59	*6.58E-02*
		60-69	*1.07E-01*			60-69	*7.54E-01*
		70+	*5.65E-01*			70+	*8.40E-01*
rs1859788	PILRB	18-29	**2.70E-04**	rs7412	CNTNAP2	18-29	*7.94E-02*
		30-39	**9.98E-14**			30-39	*5.39E-01*
		40-49	**1.77E-13**			40-49	**4.82E-03**
		50-59	**<2.22E-16**			50-59	*4.95E-01*
		60-69	**9.34E-08**			60-69	*4.99E-02*
		70+	**2.10E-05**			70+	*4.20E-02*
rs1859788	PILRA	18-29	**4.88E-03**	rs7412	SRP14	18-29	*6.17E-01*
		30-39	**1.92E-07**			30-39	*6.47E-02*
		40-49	**6.90E-06**			40-49	*3.82E-01*
		50-59	**3.65E-10**			50-59	*3.01E-01*
		60-69	**3.42E-03**			60-69	*4.20E-02*
		70+	*8.41E-02*			70+	*2.33E-01*
rs7412	LDL particle number	18-29	*3.58E-02*	rs7412	DG oag (18:1/20:4)[2]	18-29	**4.15E-03**
		30-39	**6.85E-08**			30-39	*3.32E-01*
		40-49	**6.20E-13**			40-49	**1.93E-03**
		50-59	**<2.22E-16**			50-59	**8.53E-04**
		60-69	**3.45E-04**			60-69	*2.32E-01*
		70+	*2.66E-02*			70+	*6.43E-01*
rs7412	LDL cholesterol	18-29	*0.109*	rs7412	DG oag (18:1/20:4)[1]	18-29	**1.40E-03**
		30-39	**4.48E-12**			30-39	*7.60E-01*
		40-49	**1.29E-08**			40-49	**1.12E-03**
		50-59	**2.67E-07**			50-59	**6.46E-03**
		60-69	**8.85E-04**			60-69	*4.21E-01*
		70+	*5.41E-02*			70+	*9.76E-01*
rs7412	Total cholesterol	18-29	*1.05E-01*	rs7412	DG lag (18:2/20:4) [1]	18-29	**5.96E-03**
		30-39	**9.14E-05**			30-39	*8.85E-01*
		40-49	**8.96E-06**			40-49	**1.46E-04**
		50-59	**4.62E-03**			50-59	*2.96E-02*
		60-69	*5.97E-02*			60-69	*6.96E-01*
		70+	*3.12E-01*			70+	*6.77E-01*
rs7412	LDL small	18-29	*2.41E-01*	rs7412	DG pag (16:0/20:4)[2]	18-29	**3.56E-04**
		30-39	**5.74E-03**			30-39	*7.22E-01*
		40-49	*1.00E-01*			40-49	**6.72E-04**
		50-59	**4.04E-05**			50-59	*5.53E-02*
		60-69	*1.09E-01*			60-69	*3.06E-01*
		70+	*1.09E-01*			70+	*9.02E-01*
rs7412	LDLR	18-29	*1.00E-01*	rs7412	DG oog (18:1/18:1)[1]	18-29	*2.57E-01*
		30-39	*1.69E-02*			30-39	*4.44E-01*
		40-49	**1.51E-07**			40-49	*2.26E-01*
		50-59	**1.27E-10**			50-59	*4.11E-02*
		60-69	*2.13E-01*			60-69	*6.59E-01*
		70+	*9.07E-01*			70+	*7.70E-01*
rs7412	HMOX1	18-29	*2.57E-01*	rs7412	1-Monolinolein	18-29	*4.75E-01*
		30-39	*9.35E-01*			30-39	*6.45E-01*
		40-49	**9.84E-04**			40-49	*8.41E-02*
		50-59	**8.95E-03**			50-59	**1.65E-03**
		60-69	*5.34E-01*			60-69	*8.73E-01*
		70+	*9.69E-01*			70+	*4.12E-01*

[a]For each SNP-analyte pair, Bonferroni p-value threshold for significance (in bold) was 0.05/6 age groups = 0.008

Supplementary Table 3B. SNP-analyte ANOVA p-values by age group

SNP	Analyte	Age Group	ANOVA p-value[a]	SNP	Analyte	Age Group	ANOVA p-value[a]
rs429358	LDL particle number	18-29	*1.55E-01*	rs429358	CD28	18-29	*8.68E-01*
		30-39	**1.96E-04**			30-39	**1.19E-04**
		40-49	**4.10E-06**			40-49	*4.34E-01*
		50-59	*3.20E-02*			50-59	*2.35E-01*
		60-69	*5.07E-01*			60-69	**2.49E-05**
		70+	*8.07E-01*			70+	*3.17E-01*
rs429358	LDL cholesterol	18-29	*8.42E-02*	rs429358	DG oag (18:1/20:4)[1]	18-29	*9.49E-02*
		30-39	**1.98E-03**			30-39	*1.63E-02*
		40-49	**1.40E-03**			40-49	**1.07E-06**
		50-59	*2.57E-02*			50-59	*7.71E-01*
		60-69	*4.56E-01*			60-69	*4.79E-01*
		70+	*8.89E-01*			70+	*4.31E-01*
rs429358	Total cholesterol	18-29	**6.31E-03**	rs429358	DG pag (16:1/20:4)	18-29	*5.99E-02*
		30-39	**4.83E-03**			30-39	*9.60E-03*
		40-49	*1.06E-02*			40-49	**2.82E-04**
		50-59	*3.49E-01*			50-59	*7.55E-01*
		60-69	*4.55E-01*			60-69	*8.90E-01*
		70+	*4.04E-01*			70+	*6.75E-01*
rs429358	Triglyceride:HDL ratio	18-29	*7.30E-01*	rs429358	DG oag (18:1/20:4)[2]	18-29	*2.29E-01*
		30-39	*2.70E-01*			30-39	**1.81E-03**
		40-49	**8.15E-06**			40-49	**3.11E-06**
		50-59	*7.78E-01*			50-59	*6.36E-01*
		60-69	*6.72E-01*			60-69	*2.61E-01*
		70+	*8.22E-01*			70+	*5.24E-01*
rs429358	HDL cholesterol	18-29	*2.72E-01*	rs429358 (NO CHOL-ESTEROL MEDS)	LDL particle number	18-29	**3.75E-03**
		30-39	*5.79E-01*			30-39	*8.52E-02*
		40-49	**4.32E-04**			40-49	**2.70E-03**
		50-59	*1.44E-01*			50-59	**1.75E-03**
		60-69	*9.41E-01*			60-69	*2.42E-02*
		70+	*9.10E-02*			70+	*9.41E-01*
rs429358	Triglycerides	18-29	*1.50E-01*	rs429358 (CHOL-ESTEROL MEDS)	LDL particle	18-29	*9.57E-01*
		30-39	*2.61E-01*			30-39	*2.68E-01*
		40-49	**6.49E-05**			40-49	*4.08E-01*
		50-59	*9.61E-01*			50-59	*8.32E-01*
		60-69	*6.39E-01*			60-69	*3.55E-01*
		70+	*8.13E-01*			70+	*6.96E-02*
rs429358	PLA2G7	18-29	*6.23E-01*	rs3752246	LC (d18:1/24:1)	18-29	**6.21E-04**
		30-39	*2.88E-01*			30-39	**4.23E-06**
		40-49	*2.50E-02*			40-49	**6.18E-04**
		50-59	**4.89E-04**			50-59	**7.16E-07**
		60-69	*1.65E-01*			60-69	**1.39E-04**
		70+	*8.86E-02*			70+	*3.68E-01*
rs429358	CD28	18-29	*8.68E-01*	rs3752246	LC (d18:1/16:0)	18-29	**5.89E-03**
		30-39	**1.19E-04**			30-39	*1.93E-01*
		40-49	*4.34E-01*			40-49	*1.25E-01*
		50-59	*2.35E-01*			50-59	**2.63E-03**
		60-69	**2.49E-05**			60-69	*3.05E-02*
		70+	*3.17E-01*			70+	*3.30E-01*
rs429358	DG oag (18:1/20:4)[1]	18-29	*9.49E-02*	rs3752246	DEFA1	18-29	*3.50E-01*
		30-39	*1.63E-02*			30-39	*1.26E-01*
		40-49	**1.07E-06**			40-49	*9.73E-01*
		50-59	*7.71E-01*			50-59	**5.39E-05**
		60-69	*4.79E-01*			60-69	*9.13E-02*
		70+	*4.31E-01*			70+	*6.64E-01*
rs429358	PLA2G7	18-29	*6.23E-01*	rs1093343	IDUA	18-29	*5.43E-01*
		30-39	*2.88E-01*			30-39	*7.44E-01*
		40-49	*2.50E-02*			40-49	*1.51E-01*
		50-59	**4.89E-04**			50-59	*6.54E-01*
		60-69	*1.65E-01*			60-69	**1.99E-03**
		70+	*8.86E-02*			70+	*1.39E-01*

[a]For each SNP-analyte pair, Bonferroni p-value threshold for significance (in bold) was 0.05/6 age groups = 0.008

Supplementary Table 4. SNP x sex interactions (interaction FDR p<0.1)

SNP / Nearest Gene	Analyte	Interaction Beta	Un-adjusted p_{int}	FDR-adjusted p_{int}
SNP x Sex interactions associated with proteins				
rs3851179 / PICALM	HAVCR2 (Q8TDQ0)	-0.257	1.64E-05	3.02E-02
rs3851179 / PICALM	TNFRSF4 (P43489)	-0.307	3.00E-05	3.02E-02
rs3851179 / PICALM	GIF (P27352)	-0.191	8.74E-05	4.22E-02
rs3851179 / PICALM	DSC2 (Q02487)	-0.274	1.03E-04	4.22E-02
rs3851179 / PICALM	PCOLCE (Q15113)	-0.282	1.12E-04	4.22E-02
rs3851179 / PICALM	NCR1 (O76036)	-0.268	1.26E-04	4.22E-02
rs3851179 / PICALM	TPSAB1 (Q15661)	-0.375	2.51E-04	6.08E-02
rs3851179 / PICALM	CLEC14A (Q86T13)	-0.258	2.66E-04	6.08E-02
rs3851179 / PICALM	PDCD1LG2 (Q9BQ51)	-0.077	2.73E-04	6.08E-02
rs3851179 / PICALM	ROR1 (Q01973)	-0.251	3.50E-04	7.03E-02
rs3851179 / PICALM	SPINT1 (O43278)	-0.168	4.54E-04	8.21E-02
rs3851179 / PICALM	TGFBR2 (P37173)	-0.221	5.04E-04	8.21E-02
rs3851179 / PICALM	EFEMP1 (Q12805)	-0.249	5.38E-04	8.21E-02
rs3851179 / PICALM	CD300LG (Q6UXG3)	-0.234	7.39E-04	8.21E-02
rs3851179 / PICALM	FCER2 (P06734)	-0.335	7.52E-04	8.21E-02
rs3851179 / PICALM	FSTL3 (O95633)	-0.176	7.77E-04	8.21E-02
rs3851179 / PICALM	EPHA2 (P29317)	-0.176	8.66E-04	8.65E-02
rs3851179 / PICALM	IFNGR1 (P15260)	-0.170	9.05E-04	8.65E-02
rs3851179 / PICALM	CD79B (P40259)	-0.192	1.01E-03	8.67E-02
rs3851179 / PICALM	B4GALT1 (P15291)	-0.180	1.08E-03	8.67E-02
rs3851179 / PICALM	BCAM (P50895)	-0.190	1.16E-03	8.67E-02
rs3851179 / PICALM	NOV (P48745)	-0.199	1.17E-03	8.67E-02
rs3851179 / PICALM	CD58 (P19256)	-0.169	1.20E-03	8.67E-02
rs3851179 / PICALM	CD27 (P26842)	-0.203	1.28E-03	8.67E-02
rs3851179 / PICALM	VASN (Q6EMK4)	-0.183	1.28E-03	8.67E-02
rs3851179 / PICALM	TNFRSF21 (O75509)	-0.160	1.30E-03	8.67E-02
rs3851179 / PICALM	CDH1 (P12830)	-0.248	1.61E-03	9.81E-02
rs3851179 / PICALM	CLMP (Q9H6B4)	-0.271	1.66E-03	9.81E-02
rs3851179 / PICALM	DLL1 (O00548)	-0.196	1.74E-03	9.94E-02
rs3851179 / PICALM	MFAP5 (Q13361)	-0.216	1.78E-03	9.94E-02
rs3740688 / SPI1	SMOC2 (Q9H3U7)	-0.323	4.54E-05	9.12E-02
rs3752246 / ABCA7	UBE2F (Q969M7)	0.396	2.77E-06	5.57E-03
SNP x Sex interactions associated with metabolites				
rs9473117 / CD2AP	SL palmitoyl dihydrosphingomyelin (d18:0/16:0)*	0.039	8.96E-06	1.80E-02
rs9473117 / CD2AP	SL sphingomyelin (d18:1/25:0, d19:0/24:1, d20:1/23:0, d19:1/24:0)	0.060	5.76E-05	5.78E-02
rs9473117 / CD2AP	SL palmitoyl sphingomyelin (d18:1/16:0)	0.021	1.45E-04	9.72E-02
rs9473117 / CD2AP	Plasmalogen 1-(1-enyl-palmitoyl)-2-docosahexaenoyl-GPE (P-16:0/22:6)	0.054	1.97E-04	9.86E-02
rs3851179 / PICALM	LCFA nonadecanoate (19:0)	0.040	6.73E-04	8.21E-02
rs3851179 / PICALM	LCFA oleate/vaccenate (18:1)	0.043	6.91E-04	8.21E-02
rs3851179 / PICALM	LCFA 10-nonadecenoate (19:1n9)	0.059	7.01E-04	8.21E-02
rs3851179 / PICALM	LCFA stearate (18:0)	0.032	1.16E-03	8.67E-02
rs3851179 / PICALM	LCFA margarate (17:0)	0.050	1.42E-03	9.17E-02
rs3851179 / PICALM	PUFA docosahexaenoate (DHA; 22:6n3)	0.057	1.63E-03	9.81E-02

Supplementary Table 5. ADGC datasets meta-analyzed in sex-stratified and sex-interaction analysis of rs38511179.

COHORT	Female_cases	Female_controls	Male_cases	Male_controls
ACT	333	873	199	698
ADC1	840	302	708	208
ADC2	368	106	368	50
ADC3	498	366	408	214
ADC4	168	241	136	136
ADC5	152	331	134	174
ADC6	124	225	89	113
ADC7	264	503	250	287
ADNI	113	70	155	103
GSK	379	455	287	257
MAYO	377	535	280	510
MTV	145	116	109	73
OHSU	82	84	50	69
PFIZER	374	412	322	350
ROSMAP	203	540	85	207
TARC1	199	118	124	63
TGEN2	433	177	235	188
UKS	342	87	254	83
UMVUMSSM	738	685	405	428
UPITT	790	525	465	304
WASHU1	194	113	145	74
	7116	6864	5208	4589

Supplementary Table 6. Full model results for rs3851179 by sex interaction analysis, and stratification by sex

Model	MarkerName	Allele1	Allele2	Freq1	FreqSE	MinFreq	MaxFreq	Effect	StdErr	P-value	Direction	HetISq	HetChiSq	HetDf	HetPVal
**Interaction Results**															
Model 1	11:85868640_C_T	t	c	0.3541	0.009	0.3352	0.3723	0.1157	0.0436	8.05E-03	++-++++++-+++++-+--++	0	16.404	20	0.6912
Model 2	11:85868640_C_T	t	c	0.3544	0.0091	0.3352	0.3723	0.0841	0.0478	7.84E-02	++-++++++-+++++-+---+	0	12.194	20	0.9092
**Sex-Stratified Results**															
Male Model 1	11:85868640_C_T	t	c	0.3581	0.0176	0.3193	0.3914	-0.2058	0.0353	5.62E-09	---------------+-----	0	17	20	0.6529
Male Model 2	11:85868640_C_T	t	c	0.3594	0.0171	0.3182	0.3852	-0.1758	0.0382	4.08E-06	------------+--+-?---	0	14.811	19	0.7345
Female Model 1	11:85868640_C_T	t	c	0.3539	0.0148	0.3148	0.3976	-0.0832	0.0292	4.37E-03	---+---++---+--+-----	0	18.444	20	0.5582
Female Model 2	11:85868640_C_T	t	c	0.3525	0.0142	0.3005	0.3964	-0.0868	0.0313	5.60E-03	-----+--+---++-+-----	0	15.938	20	0.7204

Supplementary Figure 1. Unadjusted box plots of clinical chemistries and metabolites significantly associated with APOE4 genotype, by age group. White boxplots=individuals who are homozygous for the major allele, gray boxplots=heterozygotes, black boxplots=minor allele homozygotes. Box plot midline=median value, lower/upper hinges=25th and 75th percentiles, respectively; lower whisker ends/upper whisker ends no further than 1.5 x interquartile range from the hinge. Data beyond whiskers are outlying points. Abbreviations/units: LDL particle number (log nmol/L); LDL cholesterol (log mg/dL); Total cholesterol (log mg/dL); HDL cholesterol (log nmol/L); Triglycerides (log nmol/L); Platelet-activating factor acetylhydrolase PLA2G7); T-cell-specific surface glycoprotein CD28 (CD28); oleoyl-arachidonoyl-glycerol (DG oag); palmitoyl-arachidonoyl-glycerol (DG pag); oleoyl-arachidonoyl-glycerol.

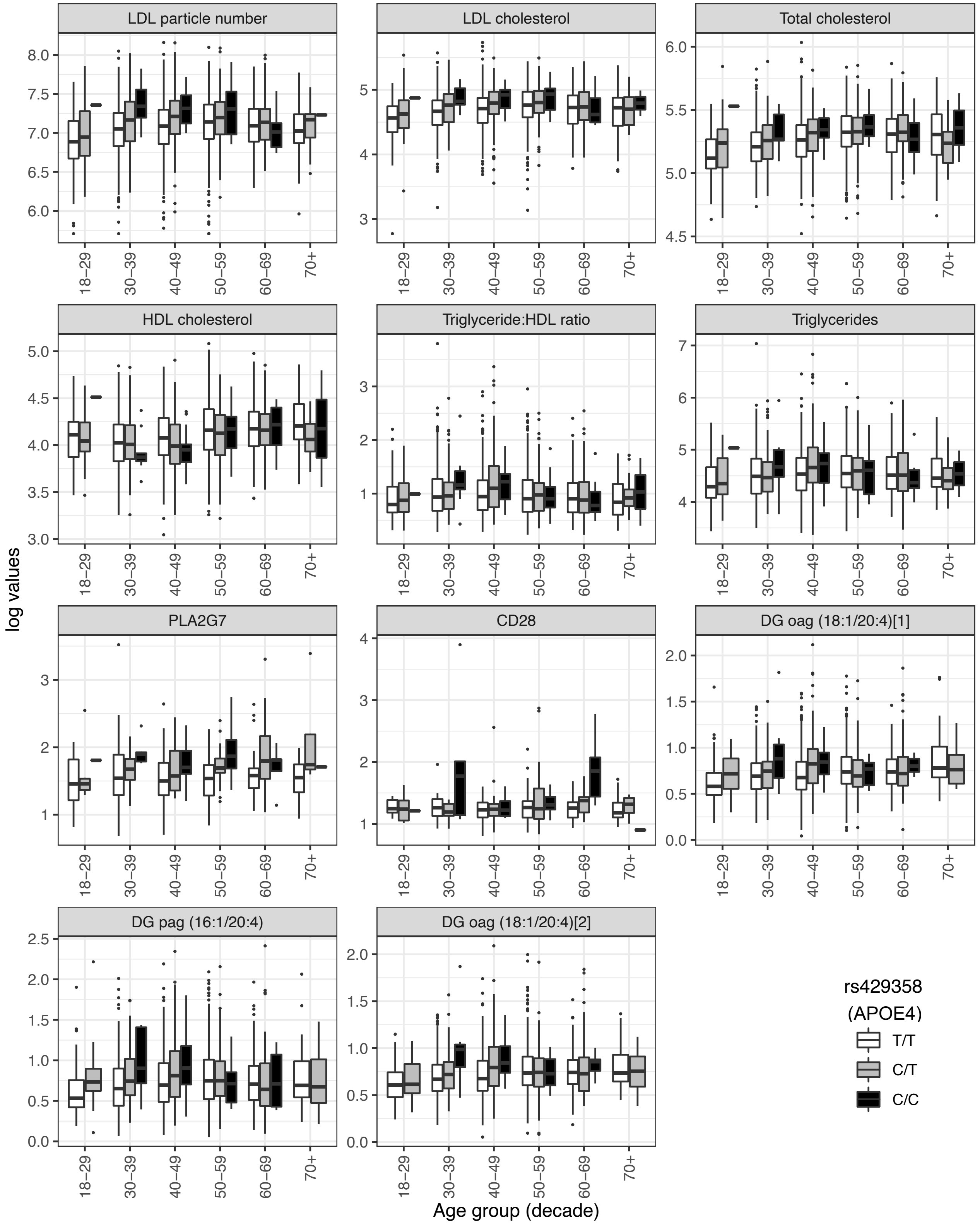

Supplementary Figure 2. LDL particle number among APOE4 genotypes, according to self-reported usage of cholesterol-lowering medications. White boxplots=individuals who are homozygous for the major allele, gray boxplots=heterozygotes, black boxplots=minor allele homozygotes. Box plot midline=median value, lower/upper hinges=25th and 75th percentiles, respectively; lower whisker ends/upper whisker ends no further than 1.5 x interquartile range from the hinge. Data beyond whiskers are outlying points. left panel shows individuals by APOE4 genotype who reported no use of a cholesterol-lowering medication; right panel shows individuals who had reported ever or currently using a cholesterol-lowering medication (individuals who did not complete this assessment survey question were not included).

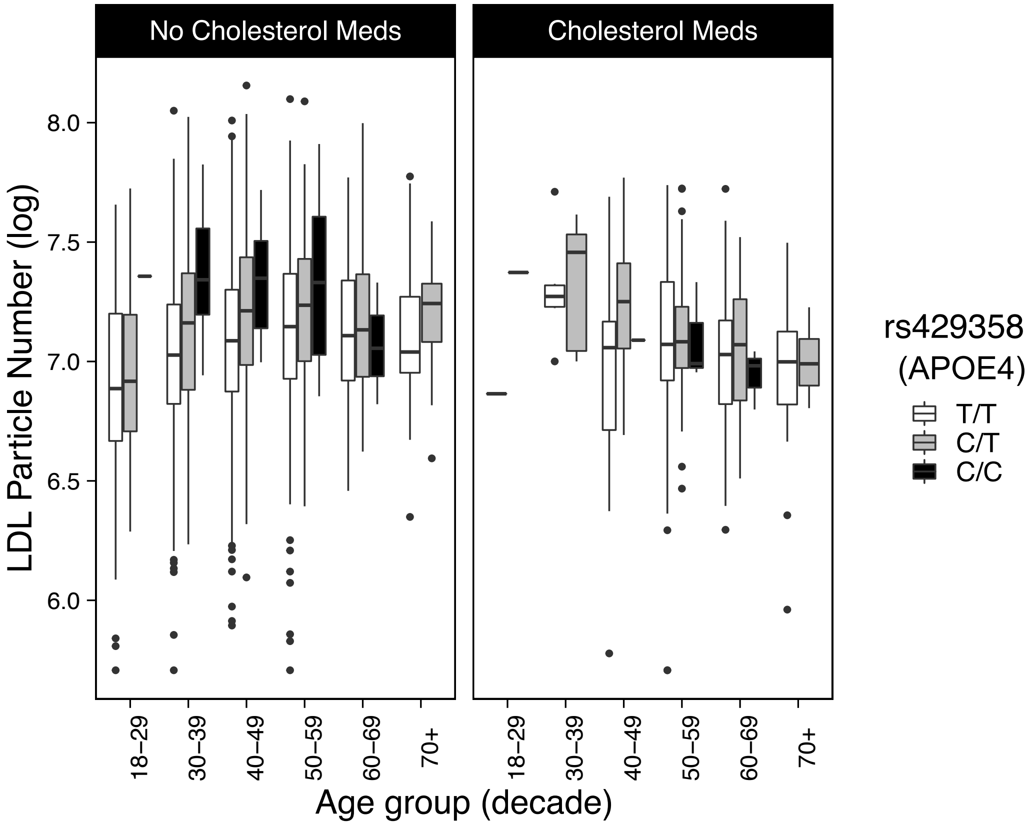

Supplementary Figure 3. Unadjusted box plots of clinical chemistries and metabolites significantly associated with APOE2 genotype, by age group. White boxplots=individuals who are homozygous for the major allele, gray boxplots=heterozygotes, black boxplots=minor allele homozygotes. Box plot midline=median value, lower/upper hinges=25th and 75th percentiles, respectively; lower whisker ends/upper whisker ends no further than 1.5 x interquartile range from the hinge. Data beyond whiskers are outlying points. Abbreviations: LDL particle number (log nmol/L); LDL cholesterol (log mg/dL); Total cholesterol (log mg/dL); LDL small (log nmol/L); Diacylglycerol, oleoyl-arachidonoyl-glycerol (DG oag); Diacylglycerol, linoleoyl-arachidonoyl-glycerol (DG lag); Diacylglycerol, palmitoyl-arachidonoyl-glycerol (DG pag); **h.** Diacylglycerol, oleoyl-oleoyl-glycerol (DG oog); Monoacelyglycerol, 1-linoleoylglycerol (1-Monolinolein).

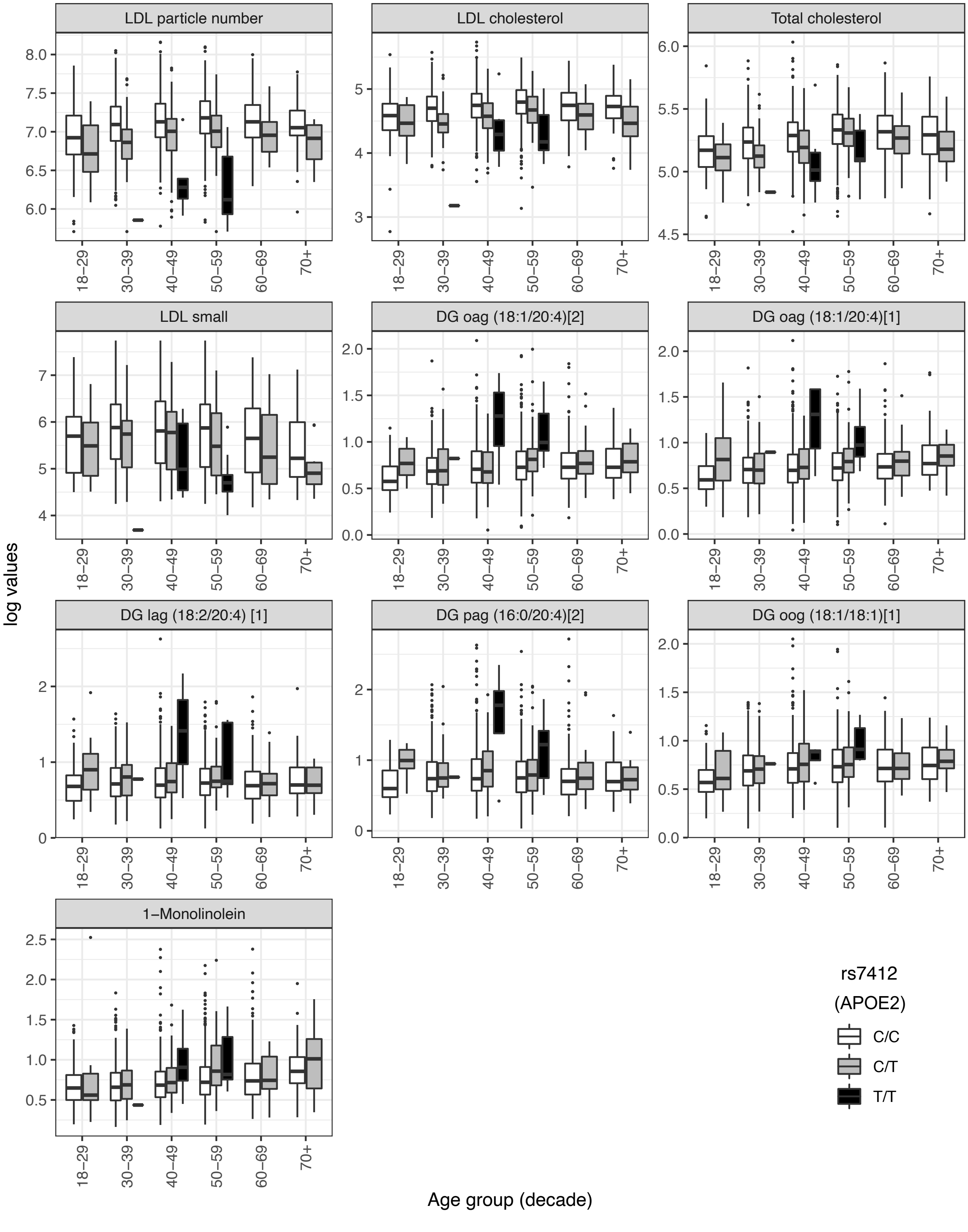

Supplementary Figure 4. Unadjusted box plots of analytes significantly associated with ABCA7, HLA-DRB1, and INPP5D genotypes, by age group. White boxplots=individuals who are homozygous for the major allele, gray boxplots=heterozygotes, black boxplots=minor allele homozygotes. Box plot midline=median value, lower/upper hinges=25th and 75th percentiles, respectively; lower whisker ends/upper whisker ends no further than 1.5 x interquartile range from the hinge. Data beyond whiskers are outlying points. Abbreviations: Sphingolipid, N-nervonoyl-sphingosine (LC (d18:1/24:1); Sphingolipid, N-palmitoyl-sphingosine (LC d18:1/16:0); Neutrophil defensin 1 (DEFA1); Alpha-L-iduronidase (IDUA).

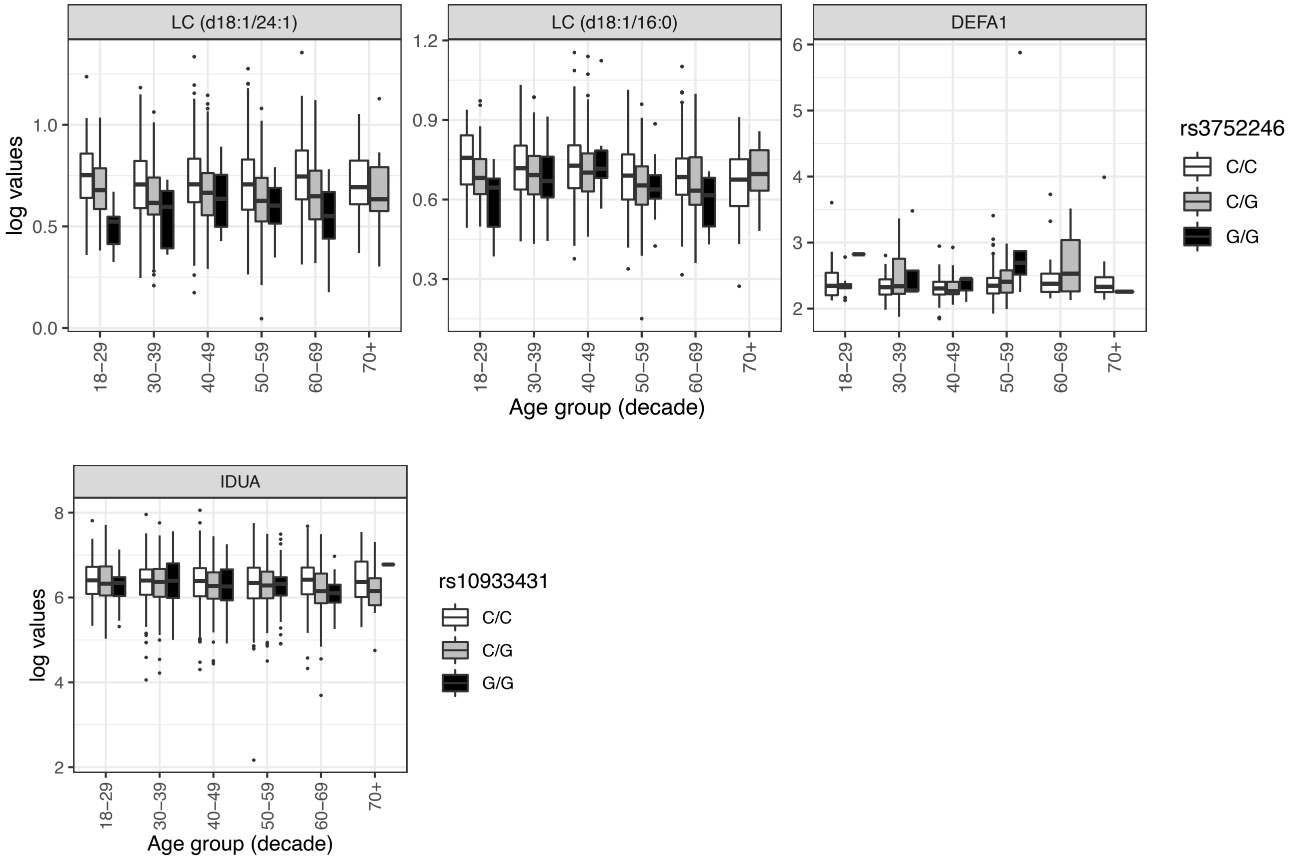

Supplementary Figure 5. Unadjusted box plots of proteins with a significant interaction term between sex and PICALM genotype at FDR<0.1, by age group. White boxplots=individuals who are homozygous for the major allele, gray boxplots=heterozygotes, black boxplots=minor allele homozygotes. Box plot midline=median value, lower/upper hinges=25th and 75th percentiles, respectively; lower whisker ends/upper whisker ends no further than 1.5 x interquartile range from the hinge. Data beyond whiskers are outlying points. For each protein, left panel shows protein levels by genotype in men; right panel shows protein levels by genotype in women.

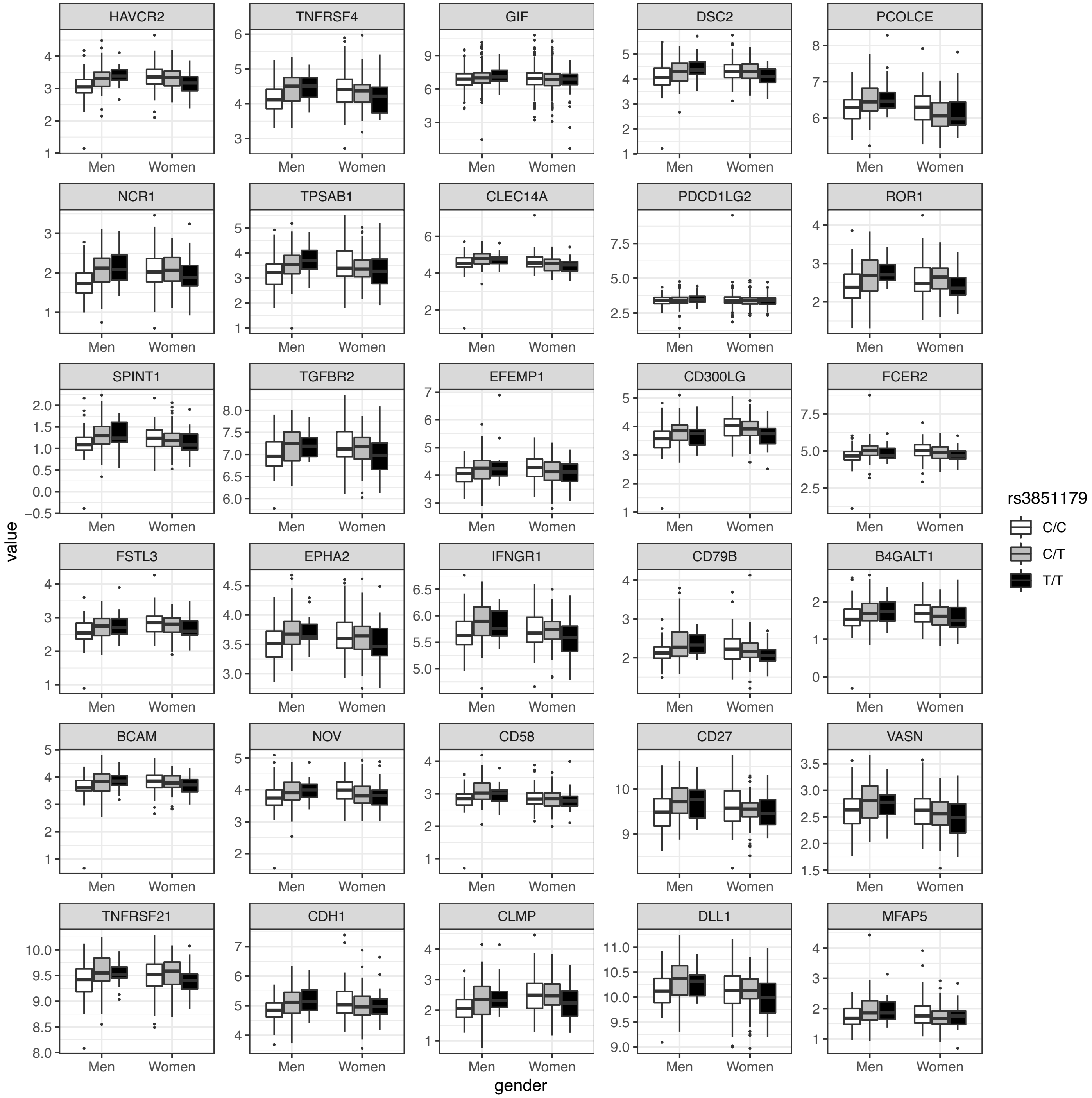

Supplementary Figure 6. Statistically significant SNP-analyte associations in self-identified Non-Hispanic white individuals only, after correcting for multiple testing (threshold FDR-adjusted p-value=0.05), by SNP. Top panel: log-transformed beta-coefficient from the linear regression model adjusted for sex, age, and genetic principal components 1-4; markers above the zero line (orange) indicate analytes that increased in value with the minor allele, while markers below the line indicate markers that decreased in value. Second panel: FDR-adjusted $-\log_{10}$ p-value; orange line at FDR-p=0.05. Proteins=red, metabolites=blue, clinical chemistries=purple. Metabolite codes: DG=diacylglycerol; LC=lactosylceramide; o=oleoyl; a=arachidonoyl; g=glycerol; l=linoleoyl; p=palmitoyl. Third panel: minor allele frequency (MAF). Bottom panel: Total sample size for each analyte-SNP regression.

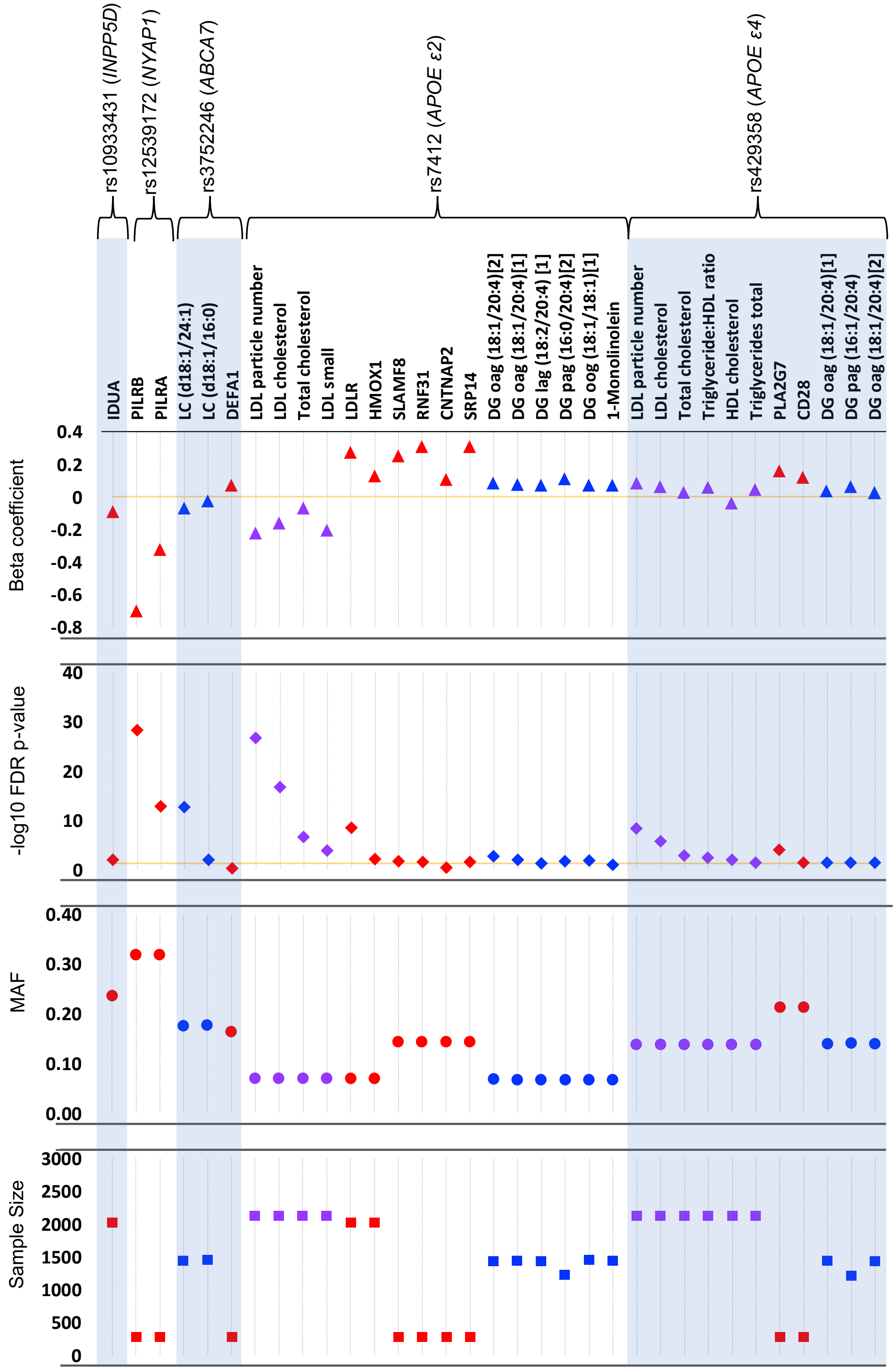

Supplementary Figure 7. Statistically significant SNP-analyte associations in individuals who did not self-identify as non-Hispanic white only, after correcting for multiple testing (threshold FDR-adjusted p-value=0.05), by SNP. Top panel: log-transformed beta-coefficient from the linear regression model adjusted for sex, age, and genetic principal components 1-4; markers above the zero line (orange) indicate analytes that increased in value with the minor allele, while markers below the line indicate markers that decreased in value. Second panel: FDR-adjusted –$\log_{10}$ p-value; orange line at FDR-p=0.05. Proteins=red, metabolites=blue, clinical chemistries=purple. Metabolite codes: DG=diacylglycerol; LC=lactosylceramide; o=oleoyl; a=arachidonoyl; g=glycerol; l=linoleoyl; p=palmitoyl. Third panel: minor allele frequency (MAF). Bottom panel: Total sample size for each analyte-SNP regression.

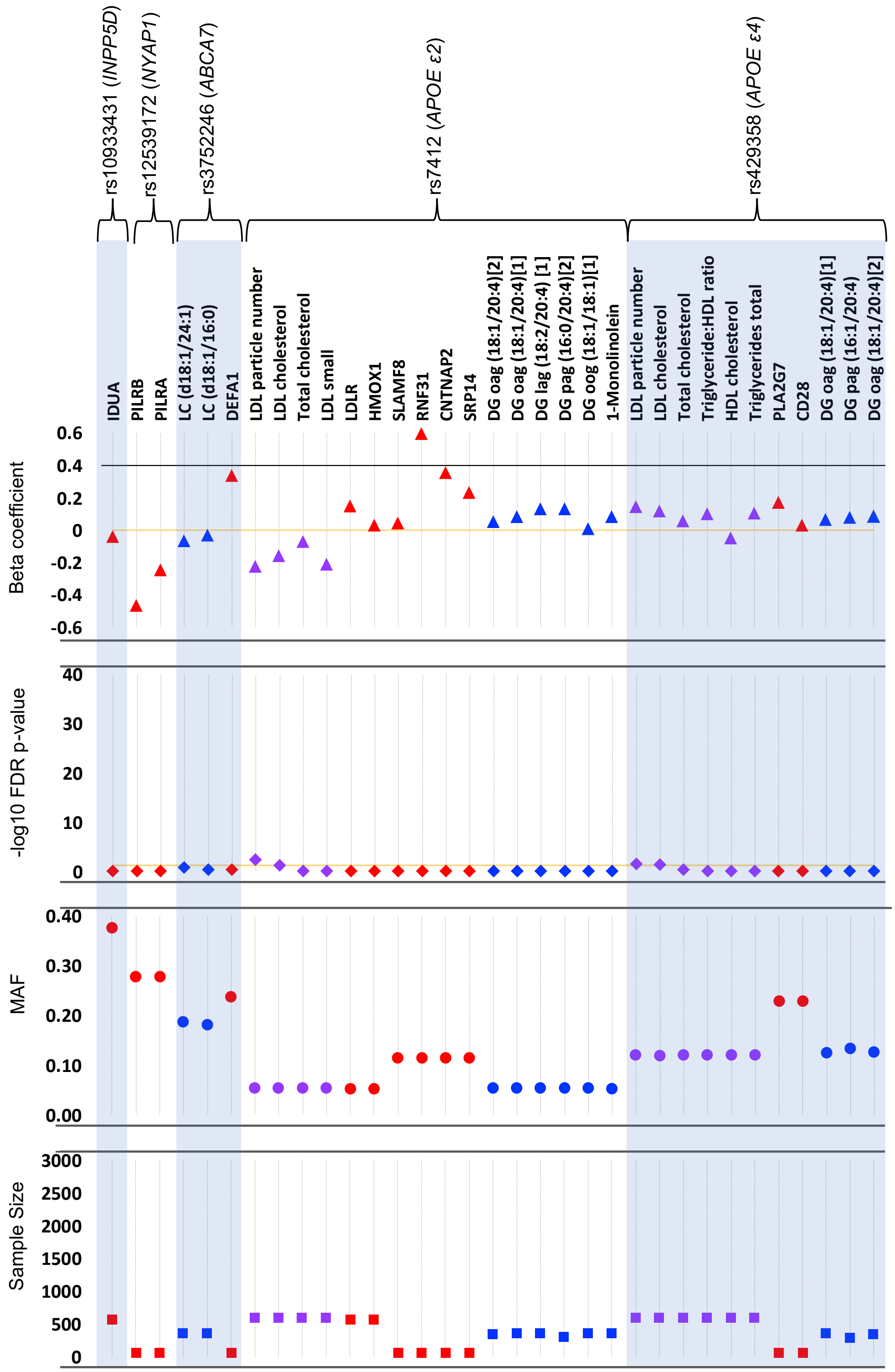

Supplementary Figure 8. Network indicating proteins with overlapping GO terms from PICALM-sex interaction analysis. Figure completed in CLUEGO, a Cytoscape plug-in.

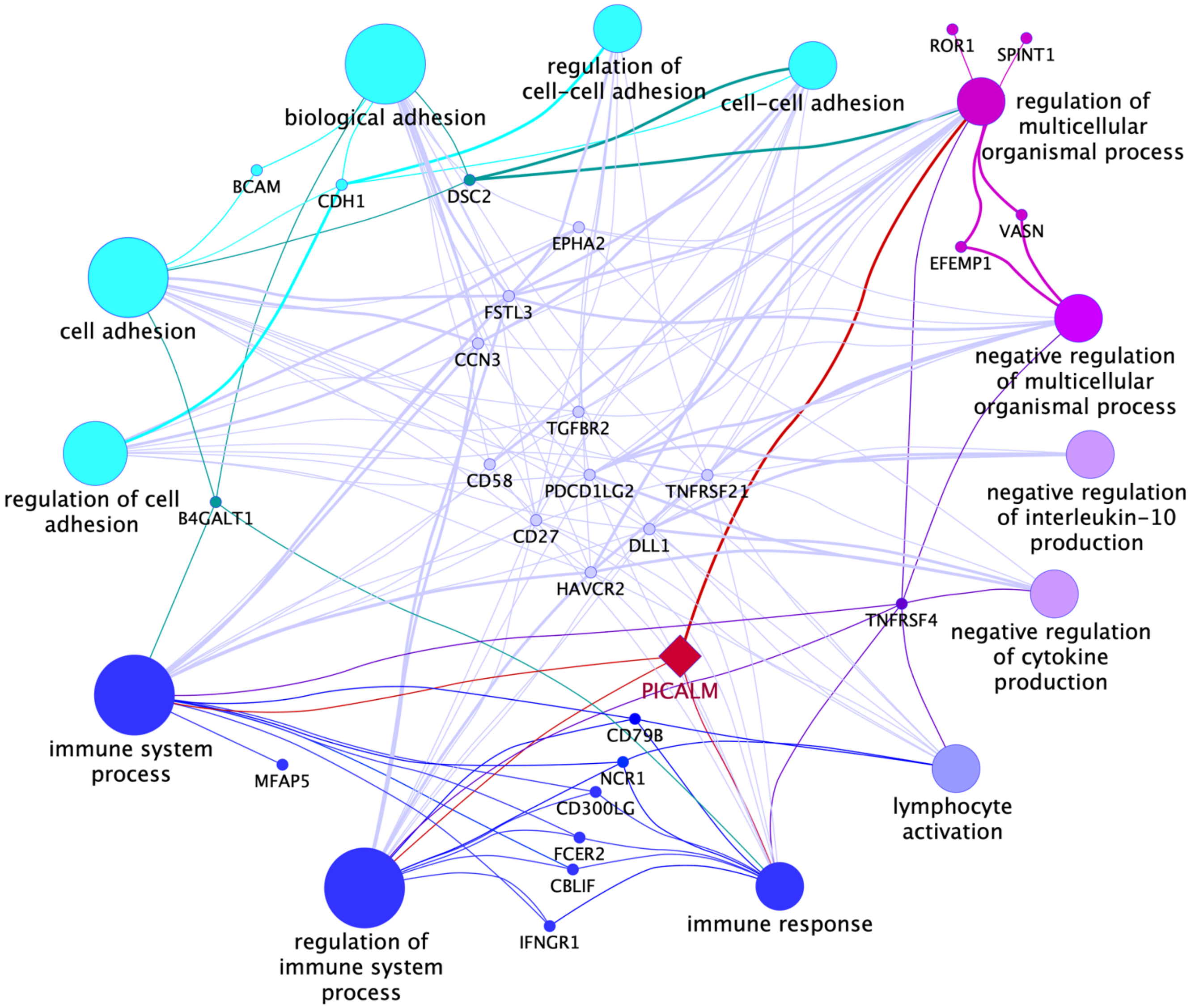

Supplement: Supplementary file 3 — Supplementary Information 3. [file 41598_2022_9825_MOESM3_ESM.pdf]
